# Selective Histone Deacetylase Inhibitor ACY-241 (Citarinostat) Plus Nivolumab in Advanced Non-Small Cell Lung Cancer: Results From a Phase Ib Study

**DOI:** 10.3389/fonc.2021.696512

**Published:** 2021-09-06

**Authors:** Mark M. Awad, Yvan Le Bruchec, Brian Lu, Jason Ye, JulieAnn Miller, Patrick H. Lizotte, Megan E. Cavanaugh, Amanda J. Rode, Calin Dan Dumitru, Alexander Spira

**Affiliations:** ^1^ Lowe Center for Thoracic Oncology and Dana-Farber Cancer Institute, Boston, MA, United States; ^2^ Bristol Myers Squibb, Princeton, NJ, United States; ^3^ Acetylon Pharmaceuticals, Inc, Boston, MA, United States; ^4^ Dana-Farber Cancer Institute and Belfer Center for Applied Cancer Science, Boston, MA, United States; ^5^ Virginia Cancer Specialists (VCS) Research Institute, Fairfax, VA, United States

**Keywords:** ACY-241, citarinostat, nivolumab, non-small cell lung cancer, HDAC6

## Abstract

**Background:**

Histone deacetylase (HDAC) overexpression has been documented in various cancers and may be associated with worse outcomes. Data from early-phase studies of advanced non-small cell lung cancer (NSCLC) suggest encouraging antitumor activity with the combination of an HDAC inhibitor and either platinum-based chemotherapy or an EGFR inhibitor; however, toxicity is a limiting factor in the use of pan-HDAC inhibitors. Selective inhibition of HDAC6 may represent a potential therapeutic target and preclinical studies revealed immunomodulatory effects with HDAC6 inhibition, suggesting the potential for combination with immune checkpoint inhibitors. This phase Ib, multicenter, single-arm, open-label, dose-escalation study investigated the HDAC6 inhibitor ACY-241 (citarinostat) plus nivolumab in patients with previously treated advanced NSCLC who had not received a prior HDAC or immune checkpoint inhibitor.

**Methods:**

The orally administered ACY-241 dose was escalated (180, 360, or 480 mg once daily). Nivolumab was administered at 240 mg (day 15 of cycle 1, then every 2 weeks thereafter). The primary endpoint was to determine the maximum tolerated dose (MTD) of ACY-241 plus nivolumab. Secondary endpoints included safety, tolerability, and preliminary antitumor activity. Pharmacodynamics was an exploratory endpoint.

**Results:**

A total of 18 patients were enrolled, with 17 patients treated. No dose-limiting toxicities (DLTs) occurred with ACY-241 at 180 or 360 mg; 2 DLTs occurred at 480 mg. The MTD of ACY-241 was 360 mg. The most common grade ≥ 3 treatment-emergent adverse events were dyspnea (n = 3; 18%) and pneumonia (n = 3; 18%). At the 180-mg dose, 1 complete response and 2 partial responses (PRs) were observed. At the 360-mg dose, 3 PRs were observed; 1 patient achieved stable disease (SD) and 1 experienced progressive disease (PD). At the 480-mg dose, no responses were observed; 1 patient achieved SD and 3 experienced PD. Acetylation analyses revealed transient increases in histone and tubulin acetylation levels following treatment. An increase in infiltrating total CD3^+^ T cells was observed following treatment.

**Conclusions:**

The study identified an MTD for ACY-241 plus nivolumab and the data suggest that the combination may be feasible in patients with advanced NSCLC. Responses were observed in patients with advanced NSCLC.

**Clinical Trial Registration:**

https://clinicaltrials.gov/ct2/show/NCT02635061 (identifier, NCT02635061).

## Introduction

Although lung cancer remains the leading cause of cancer-related death worldwide (1), recent findings of improved survival with immune checkpoint inhibitor (ICI)-based treatment regimens are likely to impact survival outcomes in patients with non-small cell lung cancer (NSCLC) (2–4). However, despite improvements in overall survival with first-line ICI monotherapy relative to platinum-based chemotherapy for programmed death ligand 1 (PD-L1)-expressing advanced NSCLC (5–7), only a minority of patients with NSCLC may respond to ICI monotherapy (8), highlighting the need to develop more effective strategies to improve the efficacy of ICIs.

Histone deacetylases (HDACs) are a family of enzymes that play a critical role in major cellular functions, including actin nucleation, cell cycle regulation, chromatin remodeling, gene splicing, and nuclear transport (9, 10). Histone deacetylase overexpression has been documented in various cancers, including gastric, lung, colorectal, and prostate, and may be associated with worse outcomes in these tumor types (11–14). Development of small-molecule HDAC inhibitors previously focused on the antiproliferative effects of HDAC inhibition by modification of gene transcription. HDAC inhibitors are approved for treatment of cutaneous and peripheral T cell lymphoma, and several others are presently under investigation (15–19). Data from phase I and II studies of advanced NSCLC have suggested encouraging antitumor activity with the combination of an HDAC inhibitor and either platinum-based doublet chemotherapy or an EGFR inhibitor (20, 21). However, toxicity is a limiting factor in the use of pan-HDAC inhibitors; for example, grade 4 thrombocytopenia was more common with vorinostat plus chemotherapy compared with chemotherapy alone in a phase II study of NSCLC (21). These results have fueled development of more selective HDAC inhibitors with improved safety profiles.

Selective inhibition of HDAC6 may represent a potential therapeutic target. Preclinical studies revealed that HDAC6 silencing or inhibition results in impaired tumor growth in xenograft mouse models using NSCLC cells (22, 23). ACY-241 (citarinostat) is an orally administered selective HDAC6 inhibitor with minimal inhibitory activity against class I and IIa HDAC enzymes. ACY-241 is hypothesized to have a more favorable safety profile than pan-HDAC inhibitors while retaining antitumor activity. Preclinical data of the structurally similar HDAC6 inhibitor ricolinostat support enhanced immunomodulation with HDAC6 inhibition (24), and preliminary data from early-phase studies in multiple myeloma showed a favorable tolerability profile of ACY-241 (25) or ricolinostat (in combination with lenalidomide and dexamethasone) (26).

The use of certain ICIs with or without platinum-doublet chemotherapy are preferred treatment options for eligible patients with advanced NSCLC [Referenced with permission from the NCCN Clinical Practice Guidelines in Oncology (NCCN Guidelines®) for NSCLC V.1.2021. © National Comprehensive Cancer Network, Inc. 2021. All rights reserved. Accessed (July 20, 2021). To view the most recent and complete version of the guideline, go online to NCCN.org. NCCN makes no warranties of any kind whatsoever regarding their content, use or application and disclaims any responsibility for their application or use in any way]. However, combining ICIs with treatments that more robustly activate the immune system may lead to greater antitumor activity. Preclinical studies in peripheral blood mononuclear cells from patients with NSCLC revealed a significant reduction in regulatory T cell (Treg) proportions coincident with CD8^+^ and CD4^+^ T cell activation upon treatment with ricolinostat compared with the class I HDAC inhibitor entinostat (24). Ricolinostat treatment also led to up-regulation of MHC class II and co-stimulatory molecules in monocytes and tumor-associated macrophages (24). Further, in an immunocompetent, genetically engineered mouse model of lung adenocarcinoma, ricolinostat treatment led to a significant elevation of CD8:Treg ratios as well as phenotypic changes resembling activated infiltrating T cells (24). Collectively, these findings suggest that HDAC6 inhibition may promote a tumor microenvironment that is amenable to ICI treatment.

The use of HDAC inhibitors in combination with immunomodulatory treatments has been the subject of investigation in several tumor types. A phase I/II trial of entinostat in combination with high-dose interleukin-2 in patients with metastatic renal cell cancer demonstrated no dose-limiting toxicities (DLTs) with hypophosphatemia, lymphopenia, and hypocalcemia being the most common grade 3 or 4 treatment-related adverse events (AEs) (27). The overall response rate with the combination was 37%, leading the authors to pursue this treatment strategy further (27). Preliminary results of a phase Ib/II trial suggest promising antitumor activity with entinostat plus pembrolizumab in patients with advanced melanoma who received prior ICI therapy; nausea, fatigue, diarrhea, and pruritus were the most common treatment-related AEs (28). In addition, studies investigating HDAC inhibition in combination with immunomodulatory treatments in patients with advanced colorectal cancer, genitourinary cancers, melanoma, uveal melanoma, or NSCLC are ongoing (29–33).

The present study investigated ACY-241 in combination with nivolumab in patients with advanced NSCLC previously treated with chemotherapy but who had not received prior HDAC or ICI therapy. The primary goal was to establish the maximum tolerated dose (MTD) of ACY-241 administered in combination with nivolumab. Secondary goals were to assesses the safety and tolerability as well as preliminary antitumor activity of the combination. To our knowledge, this is the first report of the combination of ACY-241 plus nivolumab.

## Materials and Methods

### Study Oversight

This study was approved by the institutional review boards at 4 study centers in the United States prior to commencement. This study was conducted in accordance with the Declaration of Helsinki and Good Clinical Practice Guidelines of the International Conference on Harmonisation. Informed consent was obtained from all patients prior to study entry.

### Study Design

This phase Ib, multicenter, single-arm, open-label, dose-escalation study investigated the MTD as well as the safety and preliminary antitumor activity of ACY-241 in combination with nivolumab in patients with advanced NSCLC. In this 3 + 3 study design, assessment of safety was conducted by a safety review committee (SRC) after the first 3 patients in a cohort completed 1 cycle of therapy. Dose escalation or cohort expansion could only occur with approval of the SRC. If no patient experienced a DLT (criteria defined in **Supplementary Table 1**), then dose escalation continued as planned. If 1 of 3 patients experienced a DLT, ≥ 3 additional patients could be added to the dose level. If ≤ 1 of 6 patients experienced a DLT, escalation could continue to the next cohort. If 2 DLTs were observed in up to 6 patients, the MTD was considered to have been exceeded. The MTD was defined as the highest dose level in which < 2 of 6 patients experienced a DLT. The trial is registered at clinicaltrials.gov (NCT02635061).

### Patients

Patients (≥ 18 years of age) with histologically confirmed advanced NSCLC and ≥ 1 line of prior therapy with progression or discontinuation due to toxicity were eligible for this study. Patients were required to have an Eastern Cooperative Oncology Group performance status (ECOG PS) ≤ 2 and measurable disease per Response Evaluation Criteria in Solid Tumors (RECIST) v1.1 as well as adequate hematologic and organ function. Patients who received previous HDAC inhibitor therapy and/or anti–PD-L1, or anti–cytotoxic T-lymphocyte associated protein 4 immunotherapy were not included in the study. Additionally, patients who received chemotherapy within 14 days prior to the beginning of the study were excluded.

### Treatment

**Figure 1:**
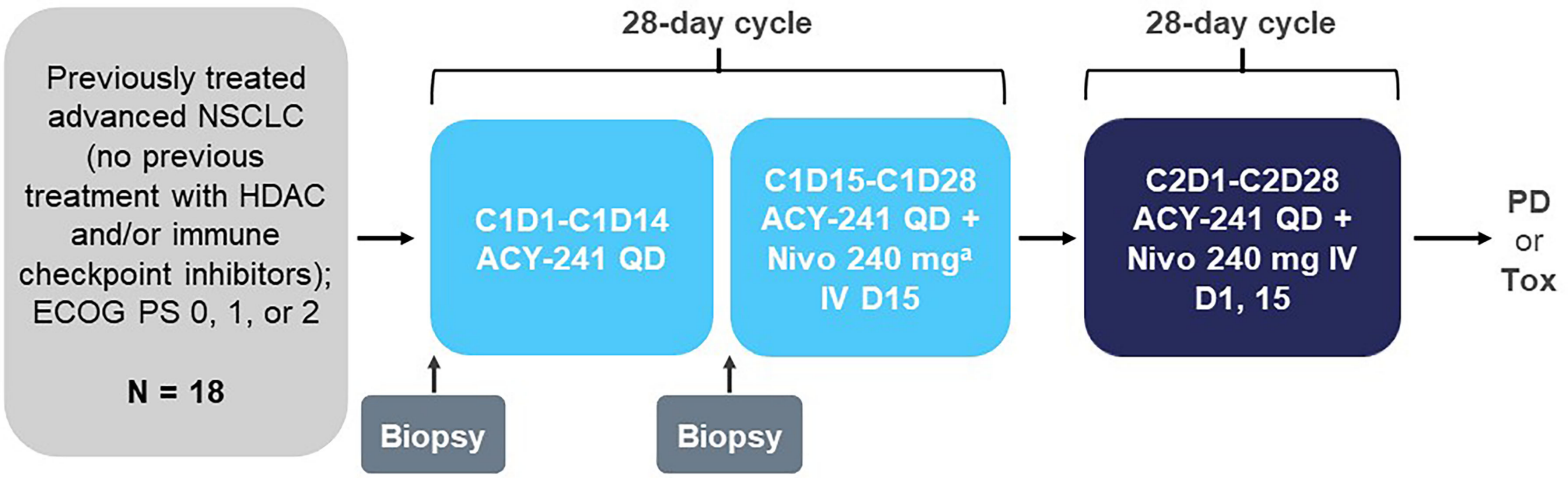
Study design. C, cycle; D, day; ECOG PS, Eastern Cooperative Oncology Group performance status; HDAC, histone deacetylase; IV, intravenous; Nivo, nivolumab; NSCLC, non-small cell lung cancer; PD, progressive disease; QD, once daily; Tox, toxicity. ^a^ First dose for the first 2 patients was 3 mg/kg prior to approval of the 240-mg dose.

Patients received orally administered ACY-241 once daily (QD) for 28 days of a 28-day treatment cycle (**Figure 1**). The initial dose of ACY-241 was 180 mg QD, which could be escalated per a modified Fibonacci sequence (180 mg QD, 360 mg QD, 480 mg QD). For cycle 1, nivolumab began on day 15 and was initially administered at 3 mg/kg by intravenous injection over 60 minutes. Starting in cycle 2, nivolumab was administered every 2 weeks on days 1 and 15. However, in September 2016, the protocol was amended to allow for a fixed 240-mg dose of nivolumab every 2 weeks following the Food and Drug Administration approval of the fixed dose. Nivolumab 3 mg/kg was administered as the first dose to the first 2 patients who were subsequently switched to the fixed dose. Patients continued the study treatment until documented progressive disease (PD) or unacceptable toxicity. Patients who experienced a DLT or other unacceptable toxicity in cycle 1 were removed from study treatment. Dose modifications were not allowed in the first dose cycle. Dose reductions reverted to the last tolerated dose for all other cycles (1 mg/kg every 14 days for nivolumab).

### Endpoints

The primary endpoint was to determine the MTD of ACY-241 in combination with nivolumab. Secondary endpoints included safety and tolerability as well as preliminary antitumor activity. Exploratory endpoints included investigation of the pharmacokinetics and pharmacodynamics of ACY-241.

### Assessments

The MTD was defined as the highest dose level at which < 2 of up to 6 patients experienced a DLT (the criteria for DLTs can be found in **Supplementary Table 1**). All grade 3/4 immune-related AEs were evaluated as potential DLTs. Safety and tolerability were measured by the proportion of patients experiencing AEs, serious AEs, DLTs, specific AEs, and AEs leading to study drug discontinuation. AEs were coded using the Medical Dictionary for Regulatory Activities, with severity assessed by the investigator according to National Cancer Institute Common Terminology Criteria for Adverse Events v4.03.

Antitumor activity was measured by disease response assessment per RECIST v1.1. Assessments of disease status were made by computed tomography (CT) or magnetic resonance imaging (MRI) at screening; day 1 of cycles 3, 5, and 8; and every 3 cycles thereafter.

Pharmacodynamic assessments were conducted using blood and tumor samples. Blood was collected at screening, cycle 1 day 15, and cycle 3 day 15.

For determination of acetylation levels, peripheral blood mononuclear cells (PBMCs) were isolated on Ficoll-Paque Plus (GE Healthcare Cat. No. 17-1440-02) and stored in 10% dimethyl sulfoxide freezing medium until analysis. Levels of acetylated histone H2 and acetylated α-tubulin were assessed by intracellular flow cytometry in the CD3^+^ subset of PBMCs. Levels of acetylation were expressed as mean fluorescence intensity (MFI) after subtraction of the MFI for the isotype control (normalized MFI).

For fluorescence-activated cell sorting immunophenotyping of peripheral blood, specimens were collected in Cyto-Chex BCT tubes (Streck Cat. No. 218980). An aliquot of each sample was taken for permeabilization as needed and analyzed with panels for T cell subsets (CD3, CD4, CD8, CD28, CD38, CD45RA, CD45RO, CD62L, and HLA-DR), regulatory T cells (CD3, CD4, CD8, CD25, CD69, and CD127), myeloid-derived suppressor cells (CD11b, CD14, and CD33), and Lin-negative cells (CD3, CD14, CD19, CD20, and CD56 [Biolegend Lineage Cocktail Cat. No. 348701]).

For circulating cytokine levels, blood was collected in EDTA tubes and cytokine analyses were performed in the plasma. An 8-plex cytokine panel (IFN-ɣ, TNF-α, interleukin [IL]-10, IL-6, IL-1b, IL-2, IL-4, and matrix metallopeptidase [MMP]-9) was evaluated on a Luminex platform.

Tumor biopsies were collected before and after 14 days of treatment with single-agent ACY-241 prior to initiating nivolumab. Tumor-infiltrating cells were separated from enzymatically dissociated tumor specimens and assessed by flow cytometry as previously described (34).

### Statistical Analyses

The target enrollment was 41 evaluable patients based on the assumption that 24 evaluable patients would be enrolled in a total of 3 dose levels plus 1 intermediate dose level in the dose-escalation part of the study, including 6 patients at the MTD, and 20 evaluable patients would be enrolled in the dose-expansion part of the study. However, the dose expansion was not undertaken, and the additional patients needed to reach the original target enrollment were not enrolled.

The DLT-evaluable population was all patients who received ≥ 80% of the target ACY-241 doses during cycle 1. The safety population was all patients who received ≥ 1 dose of study drug. The efficacy-evaluable population was all patients who met the eligibility criteria, received ≥ 1 cycle of study drug, and had ≥ 1 postbaseline efficacy assessment.

For the biomarker data, the target population was the total number of patients enrolled. Because of the limited size of the overall population, formal statistical correlative analyses were not performed. Instead, most data are displayed graphically without statistical summaries or with a select number of descriptive statistics summary measures (median, second and third quartiles, and range).

## Results

### Baseline Demographics and Clinical Characteristics

Of 18 enrolled patients, 17 were treated. Baseline characteristics of the 17 treated patients are shown in **Table 1**. The median age was 66 years (range, 42-81 years) and the majority were male (53%); 13 (76%) had an ECOG PS of 1. PD-L1 expression levels were available in 15 of the 17 cases (was not mandated at time of study onset), and the PD-L1 level was ≥ 1% in 11 patients (73% of those with known PD-L1 status). The clinical data cutoff date was July 21, 2020. At data cutoff, 2 patients remained on treatment in cycles 41 and 49 (**Supplementary Figure 1**).

**Table 1:** Baseline characteristics (safety population).

Characteristic	ACY-241 180 mg (n = 4)	ACY-241 360 mg (n = 5)	ACY-241 480 mg (n = 8)	Total (N = 17)
**Age, median (range), years**	66 (42-74)	72 (53-81)	65 (53-77)	66 (42-81)
** < 65 years, n (%)**	2 (50)	1 (20)	3 (38)	6 (35)
** ≥ 65 years, n (%)**	2 (50)	4 (80)	5 (63)	11 (65)
**Male, n (%)**	3 (75)	3 (60)	3 (38)	9 (53)
**ECOG PS, n (%)**				
** 0**	2 (50)	0	2 (25)	4 (24)
** 1**	2 (50)	5 (100)	6 (75)	13 (76)
**Histology, n (%)**				
**Adenocarcinoma**	4 (100)	3 (60)	8 (100)	15 (88)
**Squamous cell**	0	2 (40)	0	2 (12)
**Smoking status, n (%)**				
**Current/former smoker**	3 (75)	4 (80)	6 (75)	13 (76)
**Never smoked**	1 (25)	1 (20)	2 (25)	4 (24)
**PD-L1 status, n (%)**				
**≥ 1%** ^a^	1 (50)	3 (60)	7 (88)	11 (73)
**Negative** ^a^	1 (50)	2 (40)	1 (13)	4 (27)
**Unknown**	2	0	0	2
**Mutational status, n (%)**				
**Known**	4 (100)	5 (100)	6 (75)	15 (88)
***KRAS*** ^b^	0	3 (60)	4 (67)	7 (47)
***EGFR*** ^b^	0	0	1 (17)	1 (7)
**Other** ^b,c^	4 (100)	2 (40)	1 (17)	7 (47)
**Missing**	0	0	2	2
**Stage IV at baseline, n (%)**	4 (100)	5 (100)	8 (100)	17 (100)

ECOG PS, Eastern Cooperative Oncology Group performance status; PD-L1, programmed death ligand 1.

a Percentages based on patients with known status.

b Percentages based on patients with known mutations.

c Other mutations include (but are not limited to) PIK3CA, ERBB2, and BRCA2.

### Dose Escalation and MTD

No DLTs were observed with 180 or 360 mg ACY-241. Two DLTs occurred with 480 mg: 1 event of grade 3 nausea lasting > 72 hours despite medication and 1 event of grade 5 cardiac arrest in a patient who had a history of mild atrial fibrillation and mild hypertension. The grade 5 cardiac arrest event was determined to be related to ACY-241 and nivolumab. Based on these findings, 360 mg was determined to be the MTD of ACY-241.

### Safety

The most common grade ≥ 3 treatment-emergent AEs (TEAEs) were dyspnea (n = 3; 18%) and pneumonia (n = 3; 18%); other TEAEs are listed in **Table 2**. Of note, 1 event of grade 3 cerebrovascular accident occurred due to brain metastasis and was considered unrelated to study drug. Treatment discontinuation due to AEs occurred in 2 patients (11%).

**Table 2:** Safety (n = 17; safety population).

TEAE, n (%)^a^	Grade 1/2	Grade ≥ 3
**Dyspnea**	2 (11.8)	3 (17.6)
**Pneumonia**	0	3 (17.6)
**Diarrhea**	1 (5.9)	2 (11.8)
**Chronic obstructive pulmonary disease**	0	2 (11.8)
**Presyncope**	0	2 (11.8)
**Fatigue**	6 (35.3)	1 (5.9)
**Decreased appetite**	4 (23.5)	1 (5.9)
**Nausea**	3 (17.6)	1 (5.9)
**Upper respiratory tract infection**	3 (17.6)	1 (5.9)
**Anemia**	2 (11.8)	1 (5.9)
**Abdominal pain**	1 (5.9)	1 (5.9)
**Hyperglycemia**	1 (5.9)	1 (5.9)
**Cardiac arrest**	0	1 (5.9)^b^
**Cerebrovascular accident**	0	1 (5.9)
**Myasthenia gravis**	0	1 (5.9)^b^
**Respiratory syncytial virus infection**	0	1 (5.9)
**Respiratory tract infection**	0	1 (5.9)
**Sciatica**	0	1 (5.9)
**Weight increased**	0	1 (5.9)

TEAE, treatment-emergent adverse event.

a TEAEs ordered first by incidence of grade ≥ 3, then by incidence of grade 1/2.

b Grade 5 TEAE.

The grade 3 cerebrovascular accident occurred in a patient with a history of gastroesophageal reflux disease, Crohn’s disease, pulmonary fibrosis, diarrhea, and insomnia. In the opinion of the investigator, the grade 3 cerebrovascular accident was unrelated to ACY-241 and possibly related to disease progression. One event of grade 5 myasthenia gravis, determined to be related to ACY-241 and nivolumab, occurred at the 360-mg dose in a patient with a history of moderate fatigue.

### Antitumor Activity

**Figure 2:**
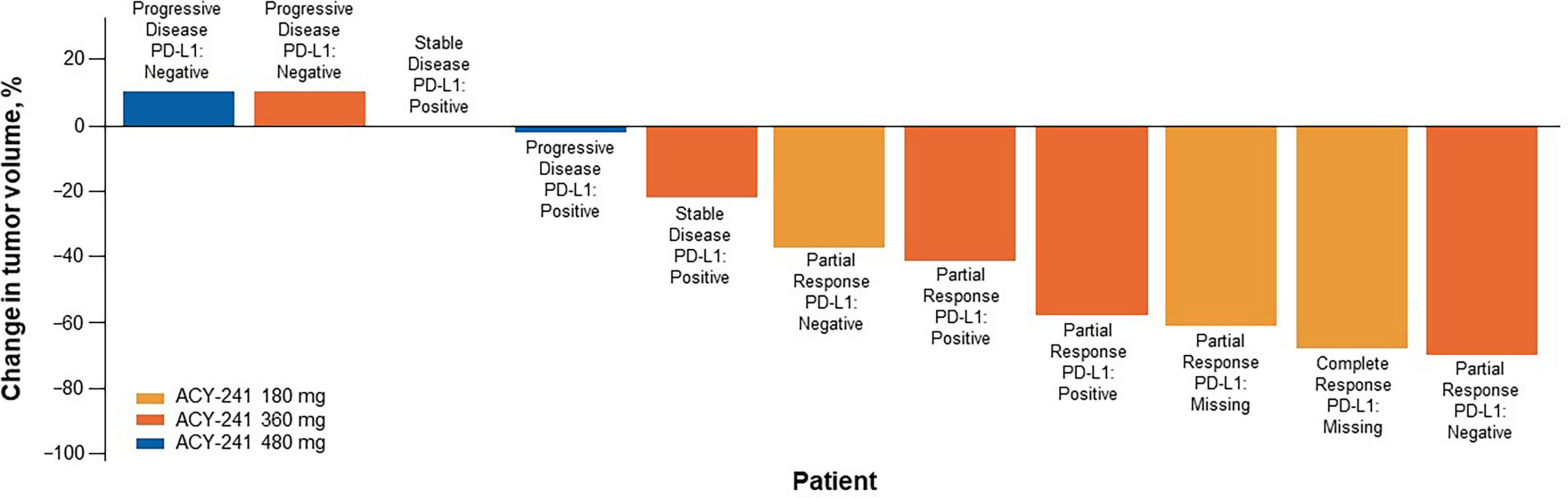
Sum of tumor shrinkage and best response per RECIST v1.1 (n = 11). Tumor shrinkage measured from baseline. One response-evaluable patient received first dose of nivolumab but had progressive disease before first tumor assessment and was excluded here. PD-L1, programmed death ligand 1; RECIST, Response Evaluation Criteria in Solid Tumors.

At the 180-mg dose, 1 complete response (CR) and 2 partial responses (PRs) were observed (**Table 3**). At the 360-mg dose, 3 PRs were observed, 1 patient achieved stable disease (SD), and 1 experienced PD. At the 480-mg dose, no response was observed, 1 patient achieved SD, and 3 experienced PD. Among the 11 response-evaluable patients, 8 had tumor shrinkage (**Figure 2**). Among the 6 patients with either a CR or PR, PD-L1 status was negative in 2 (1 PR each for 180 and 360 mg).

**Table 3:** Best response per RECIST v1.1 (intent-to-treat population).

Response, n (%)	ACY-241 180 mg (n = 4)	ACY-241 360 mg (n = 5)	ACY-241 480 mg (n = 9)	Total (N = 18)
**Best response**				
**Complete response**	1 (25)	0	0	1 (6)
**Partial response**	2 (50)^a^	3 (60)^a^	0	5 (28)
**Stable disease**	0	1 (20)^c^	1 (11)	2 (11)
**Progressive disease**	0	1 (20)	3 (33)	4 (22)
**Not evaluable**	1 (25)^b^	0	5 (56)^b^	6 (33)
**Overall response rate**	3 (75)	3 (60)^a^	0	6 (33)

PD-L1, programmed death ligand 1; RECIST, Response Evaluation Criteria in Solid Tumors.

a One patient with known PD-L1 status was PD-L1 negative.

b Patient discontinued before tumor assessment or did not reach first postbaseline tumor assessment. One patient withdrew consent, 2 patients had progressive disease before nivolumab treatment, 2 patients discontinued due to adverse events, and 1 patient died before the first tumor assessment.

c One patient had an unconfirmed partial response before progression.

### Pharmacodynamics

Although ACY-241 is a potent inhibitor of HDAC6 (a class IIb HDAC), it does retain its inhibitory activity against class I HDACs (ie, HDAC1, HDAC2, HDAC3, and HDAC8). Analysis of acetylation levels revealed transient increases in histone (class I target) and tubulin (HDAC6 target) acetylation levels following treatment (**Supplementary Figure 2**). No clear separation of measured acetylation levels was observed between the different dose levels, although at cycle 3, histone acetylation appeared to reach lower peaks. There were no clear trends in levels of serum cytokines, except for slight increases in levels of serum IL-1β, MMP-9, and possibly IL-10 at the analysis on cycle 3 day 15 (ie, after administration of nivolumab; **Supplementary Figure 3**). Flow cytometry of peripheral immune cells showed no clinically meaningful changes in levels of different T cell subpopulations following treatment with ACY-241 (**Supplementary Figure 4**).

**Figure 3:**
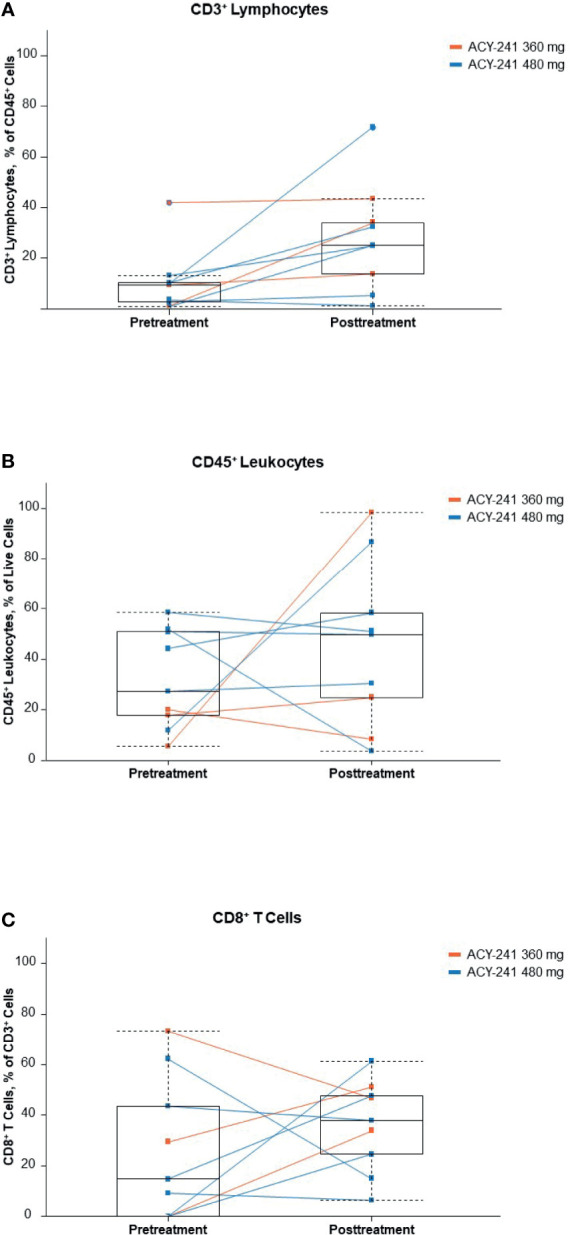
Infiltrating immune cells **(A)**, CD3^+^ lymphocytes; **(B)**, CD45^+^ leukocytes; **(C)**, CD8^+^ T cells. Percentages of selected immune cell subpopulations in tumor biopsies before and after ACY-241 treatment. These patients originated in 2 dose-level cohorts, which are indicated by different colors (orange, 360 mg; blue, 480 mg). To facilitate the determination of trends, a light-blue box plot (median, second, and third quartiles, and min-max range) is overlaid on the line plots.

Immune profiling of paired tumor samples was completed in 9 patients and allowed comparison of the pretreatment and post-ACY-241 treatment tumor microenvironment. With 1 exception in a patient who had a repeat biopsy 3 days after initiation of nivolumab treatment, the comparison describes the effects of ACY-241 monotherapy. Levels of tumor-infiltrating regulatory T cells, natural killer cells, myeloid-derived suppressor cells, and macrophages varied between patients with no obvious dose or treatment trends (**Supplementary Figure 5**). However, there was an increase in infiltrating total CD3^+^ T cells and a discernable, albeit less pronounced, increase in CD8^+^ cytotoxic T cells observed following treatment (**Figure 3**).

## Discussion

Data from early-phase studies of advanced NSCLC have suggested encouraging antitumor activity with the combination of HDAC inhibitors and chemotherapy (carboplatin plus paclitaxel) or an EGFR inhibitor; however, toxicity is a limiting factor in the use of pan-HDAC inhibitors (20, 21). The current phase Ib study found no DLTs with 180 or 360 mg ACY-241 plus nivolumab. In the current study, the MTD of ACY-241 was determined to be 360 mg. It is important to note that, although no DLTs were identified with the 360-mg dose, 1 event of grade 5 myasthenia gravis occurred at the 360-mg dose and was determined to be related to ACY-241 and nivolumab. This patient reported grade 3 fatigue that resolved, followed by grade 3 myasthenia at 12 weeks of treatment, which worsened to grade 5 after 1 week despite medical interventions. One other treatment-emergent grade 5 event occurred in a patient who experienced cardiac arrest. This patient was treated with the 480-mg ACY-241 dose for 5 weeks, and the patient’s death was also determined to possibly be related to ACY-241 and nivolumab treatment.

The disease control rate was 80% (3 PRs and 1 SD) in the 5 patients who received 360 mg ACY-241 in combination with nivolumab; however, only 1 of 9 patients who received 480 mg ACY-241 achieved SD as a best response. Although these numbers are small, the results may suggest that the effect of ACY-241 plateaued at the 360-mg dose and saturated at the 480-mg dose. Several studies have demonstrated that possibly depending on the dose used, HDAC inhibitors can have pro- or anti-inflammatory effects, which may determine their antitumor activity (reviewed in Kroesen et al. and Hull et al.) (35, 36). Regarding the current study, a potential explanation for the lack of dose response beyond 360 mg may be an immunosuppressive effect with the 480-mg dose, leading to a potentially detrimental treatment effect in an already immunosuppressed tumor microenvironment. A total of 17 patients were treated in the present analysis; however, a target enrollment of 41 patients was planned. Although the tumor response data were encouraging, an expansion of patients was not undertaken because in order to determine a true effect, a larger randomized trial would be needed.

Biomarker analyses showed transient increases in histone and tubulin acetylation, increased cytokine levels, increases in tumor-infiltrating cytotoxic T cells, and decreases in tumor-infiltrating natural killer cells following treatment. The increased tubulin acetylation supports the HDAC6 inhibitory effects of ACY-241; however, the increased histone acetylation suggests that, to some extent, pan-HDAC activity is occurring at the tested doses. The hypothesis for conducting the current study was to enhance the antitumor activity through increased immune system tumor recognition by ACY-241 in combination with the immune checkpoint blockade activity of nivolumab. In the current study, an increase in cytotoxic T cells following treatment was observed. The reinvigoration of T cell effector function upon nivolumab treatment (37) may be accentuated by increasing the number of infiltrating cytotoxic T cells, which, in turn, may increase antitumor activity. However, the small numbers and somewhat variable responses limit the conclusions that can be drawn in this regard.

The primary goal of this study was to establish the MTD of ACY-241 administered in combination with nivolumab. Secondary goals were to assesses the safety and tolerability as well as preliminary antitumor activity of the combination. As is the case in early-phase studies, the small sample size as well as the lack of comparator arm precludes any contextualization with efficacy outcomes established elsewhere. In addition, nivolumab monotherapy has demonstrated efficacy in patients with previously treated NSCLC (38, 39); therefore, it may be difficult to discern whether there was any added benefit with the combination.

Patients in this study had received ≥ 1 prior line of therapy other than anti–PD-(L)1. Certain PD-(L)1 immunotherapies are preferred first-line therapy options for eligible patients with advanced NSCLC, but other first-line immunotherapies are also recommended [Referenced with permission from the NCCN Clinical Practice Guidelines in Oncology (NCCN Guidelines®) for NSCLC V.1.2021. © National Comprehensive Cancer Network, Inc. 2021. All rights reserved. Accessed (July 20, 2021). To view the most recent and complete version of the guideline, go online to NCCN.org. NCCN makes no warranties of any kind whatsoever regarding their content, use or application and disclaims any responsibility for their application or use in any way]. Analysis of ACY-241 and nivolumab as a first-line treatment or in patients who have received a prior PD-(L)1 therapy could elucidate the efficacy of this combination in a larger patient population.

## Conclusions

The study identified an MTD for ACY-241 in combination with nivolumab and the data suggest that the combination may be feasible in patients with advanced NSCLC. The tumor response data were encouraging. However, given the totality of the safety profile, further research may be needed to understand the use of this combination as a potential treatment option for patients with NSCLC.

## Data Availability Statement

The original contributions presented in the study are included in the article/**Supplementary Material**, further inquiries can be directed to the corresponding author. Data requests may be submitted to Celgene, a Bristol-Myers Squibb Company at https://vivli.org/ourmember/celgene/ and must include a description of the research proposal.

## Ethics Statement

All relevant ethical approvals from institutional review board/independent ethics committee have been obtained prior to study commencement. Written informed consent was obtained from all patients prior to study entry.

## Author Contributions

Substantial contributions to the conception or design of the work: MA, YLB, BL, and JM. Acquisition, analysis, or interpretation of data for the work: MA, YLB, BL, JY, JM, PL, MC, AR, CD, and AS. Drafting the work or revising it critically for important intellectual content: MA, YLB, BL, JY, JM, PL, MC, AR, CD, and AS. Final approval of the version to be published: MA, YLB, BL, JY, JM, PL, MC, AR, CD, and AS. Agreement to be accountable for all aspects of the work in ensuring that questions related to the accuracy or integrity of any part of the work are appropriately investigated and resolved: MA, YLB, BL, JY, JM, PL, MC, AR, CD, and AS. All authors contributed to the article and approved the submitted version.

## Funding

The authors declare that this study received funding from Celgene Corporation, a wholly owned subsidiary of Bristol Myers Squibb.

## Conflict of Interest

MA: Consulting, Bristol Myers Squibb, AstraZeneca, Achilles, AbbVie, Neon, Maverick, Nektar, Hegrui, Syndax, Gritstone; Research funding, Bristol Myers Squibb, Genentech, Lilly, AstraZeneca. YLB, BL, JM, and CD: employment, Bristol Myers Squibb. BL, JM, and CD: stock, Bristol Myers Squibb. JY: employment, Acetylon Pharmaceuticals. Celgene Corporation was involved in the study design, collection, analysis, interpretation of data, and funded the writing of this article.

The remaining authors declare that the research was conducted in the absence of any commercial or financial relationships that could be construed as a potential conflict of interest.

## Publisher’s Note

All claims expressed in this article are solely those of the authors and do not necessarily represent those of their affiliated organizations, or those of the publisher, the editors and the reviewers. Any product that may be evaluated in this article, or claim that may be made by its manufacturer, is not guaranteed or endorsed by the publisher.
