# Corrigendum: Selective Histone Deacetylase Inhibitor ACY-241 (Citarinostat) Plus Nivolumab in Advanced Non-Small Cell Lung Cancer: Results From a Phase Ib Study

**DOI:** 10.3389/fonc.2022.889996

**Published:** 2022-06-07

**Authors:** Mark M. Awad, Yvan Le Bruchec, Brian Lu, Jason Ye, Julie Ann Miller, Patrick H. Lizotte, Megan E. Cavanaugh, Amanda J. Rode, Calin Dan Dumitru, Alexander Spira

**Affiliations:** ^1^Lowe Center for Thoracic Oncology and Dana-Farber Cancer Institute, Boston, MA, United States; ^2^Bristol Myers Squibb, Princeton, NJ, United States; ^3^Acetylon Pharmaceuticals, Inc, Boston, MA, United States; ^4^Dana-Farber Cancer Institute and Belfer Center for Applied Cancer Science, Boston, MA, United States; ^5^Virginia Cancer Specialists (VCS) Research Institute, Fairfax, VA, United States

**Keywords:** ACY-241, citarinostat, nivolumab, non-small cell lung cancer, HDAC6

Angeliki Zarotiadou was not included in the Acknowledgments section in the published article. The corrected Acknowledgments section appears below.

“The authors thank the patients who participated in the study and their families. Additional thanks to Precision for Medicine, formerly ApoCell, for serum cytokine and blood immunophenotyping. Biostatistics assistance was provided by Angeliki Zarotiadou, MSc. Writing assistance was provided by Aaron Runkle, PhD, CMPP, of MediTech Media, Ltd, and funded by Bristol Myers Squibb. The authors presented these results in an abstract and poster at the 2019 ASCO Annual Meeting; May 31-June 4, 2019; Chicago, IL (40)”.

In the original article, the reference for NCCN Guidelines^®^ for NSCLC V.1.2021 was incorrectly written in the in-text citation as “[Referenced with permission from the NCCN Clinical Practice Guidelines in Oncology (NCCN Guidelines^®^) for NSCL V.1.2021. ^©^ National Comprehensive Cancer Network, Inc. 2021. All rights reserved. Accessed (December 3, 2020). To view the most recent and complete version of the guideline, go online to NCCN.org. NCCN makes no warranties of any kind whatsoever regarding their content, use or application and disclaims any responsibility for their application or use in any way]”.

It should be “[Referenced with permission from the NCCN Clinical Practice Guidelines in Oncology (NCCN Guidelines^®^) for NSCLC V.1.2021. ^©^ National Comprehensive Cancer Network, Inc. 2021. All rights reserved. Accessed (July 20, 2021). To view the most recent and complete version of the guideline, go online to NCCN.org. NCCN makes no warranties of any kind whatsoever regarding their content, use or application and disclaims any responsibility for their application or use in any way]”.

The authors apologize for these errors and state that this does not change the scientific conclusions of the article in any way. The original article has been updated.

## Publisher’s Note

All claims expressed in this article are solely those of the authors and do not necessarily represent those of their affiliated organizations, or those of the publisher, the editors and the reviewers. Any product that may be evaluated in this article, or claim that may be made by its manufacturer, is not guaranteed or endorsed by the publisher.

