# Supplementary material for: Selective Histone Deacetylase Inhibitor ACY-241 (Citarinostat) Plus Nivolumab in Advanced Non-Small Cell Lung Cancer: Results From a Phase Ib Study
Source: Front Oncol. 2021 Sep 6;11:696512. doi: 10.3389/fonc.2021.696512 (PMC8451476; doi:10.3389/fonc.2021.696512)
Supplement: Supplementary file 1 [file DataSheet_1.docx]

Supplementary Material

**Supplementary Table 1.** Criteria for defining DLTs (if determined to be related to ACY-241)

| Toxicity | Criteria |
| --- | --- |
| Hematologic | Grade 4 neutropenia (ANC < 500/μL) lasting > 7 days^a^ |
|  | Febrile neutropenia (ANC < 1000/μL with a single temperature > 38.3°C [101.0°F] or 38.0°C [100.4°F] lasting > 1 hour) |
|  | Grade 4 thrombocytopenia (platelet count < 25,000/μL) |
|  | Grade 3 thrombocytopenia (platelet count 25,000-50,000/μL) with clinically important bleeding |
| Nonhematologic^b^ | Grade ≥ 3 nausea, vomiting, and/or diarrhea lasting > 72 hours despite maximal medical intervention |
|  | AST and ALT > 2 × increase over baseline lasting > 72 hours |
|  | Delay of patient continuing to cycle 2 by > 7 days due to AEs related to ACY-241 |
| Immune related^c^ | Immune-related toxicities among patients treated with nivolumab have clustered among skin, gastrointestinal, endocrine, and liver-related events as follows: |
|  | Skin: alopecia, dry skin, hyperhidrosis, night sweats, pruritus, rash/desquamation, toxic epidermal necrolysis, urticaria, vitiligo |
|  | Gastrointestinal: abdominal discomfort or pain, anal ulcer, colitis (including ulcerative, hemorrhagic, ischemic, and megacolon), constipation, cramping, diarrhea (including hemorrhagic), diverticulitis/diverticulum, duodenitis, dyspepsia, dysphagia, enteritis, esophagitis, gastritis (including erosive), gastrointestinal hemorrhage (including rectal), hematochezia, ileitis, ileus, intestinal obstruction, intestinal perforation (including small and large intestines), melena, nausea, pancreatitis, peritonitis, stomatitis (including aphthous), vomiting, vasculitis gastrointestinal |
|  | Endocrine: adrenal insufficiency (including Addison disease), glucose tolerance impaired, hyperthyroidism, hypogonadism, hypophysitis/hypopituitarism (autoimmune), hypothyroidism, pituitary enlargement, thyroiditis (autoimmune) |
|  | Liver: hepatitis, jaundice |
| Other | Grade 3 and 4 laboratory investigations, excluding cardiac function tests, may be evaluated by the SRC to determine whether dose cohorts should be expanded |

AE, adverse event; ALT, alanine aminotransferase; ANC, absolute neutrophil count; AST, aspartate aminotransferase; DLT, dose-limiting toxicity; SRC, safety review committee.

^a^ If neutropenia is attributed to ACY-241, ANC is to be repeated on day 6 to determine whether it is a DLT.

^b^ All other grade 3 or 4 nonhematologic toxicities will be considered as potential DLTs.

^c^ All grade 3 or 4 immune-related toxicities will be considered as potential DLTs.

**Supplementary Figure 1.** Patient status.


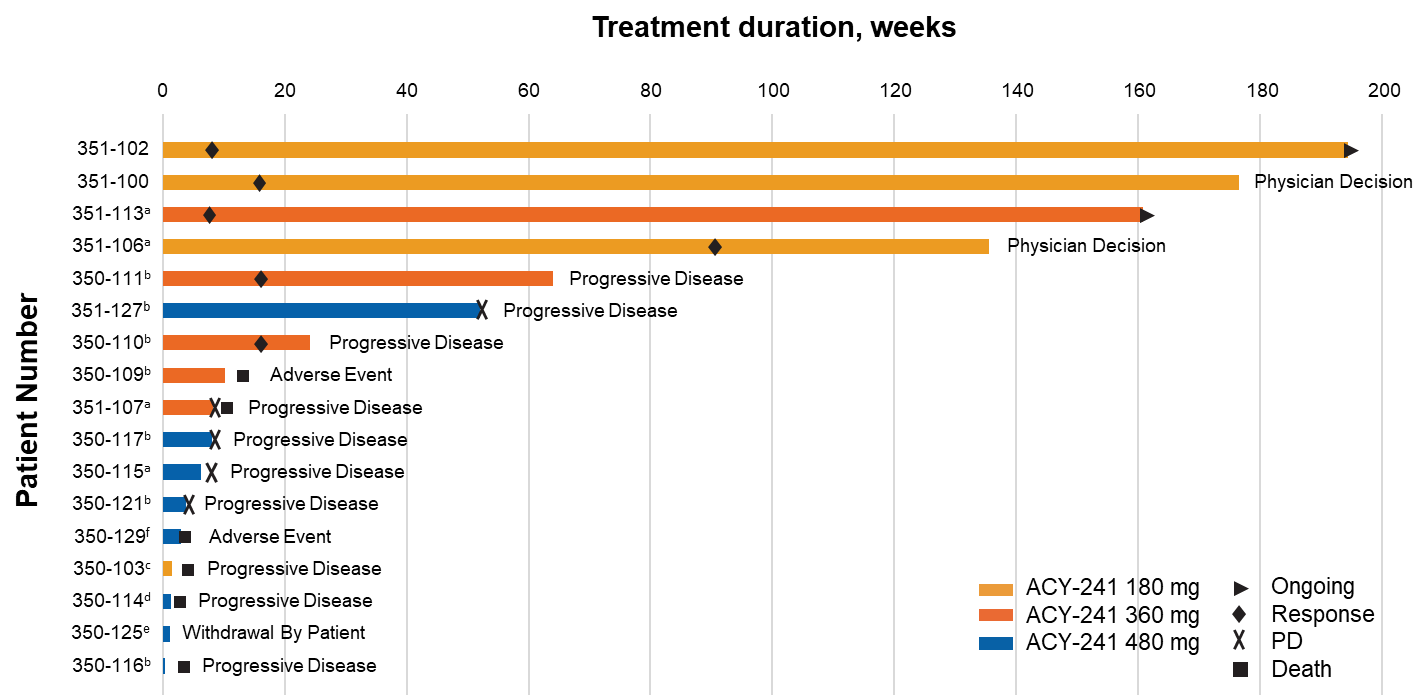


AE, adverse event; PD, progressive disease; PD-L1, programmed death ligand 1; PR, partial response.

^a^ PD-L1 negative.

^b^ PD-L1 positive, *KRAS* mutation.

^c^ PD-L1 positive, *PIK3CA* mutation.

^d^ PD-L1 positive, *EGFR* mutation.

^e^ PD-L1 positive, *ERBB2* *BRAC2* mutation.

^f^ PD-L1 positive, mutation status missing.

**Supplementary Figure** **2.** Histone **(A)** and tubulin **(B)** acetylation. Measured levels of substrates for class I HDAC and HDAC6 after administration of ACY-241, expressed as normalized mean fluorescence intensity. Each line graph represents an individual patient, and the dose level (or cohort) each patient was assigned to is indicated by a different color (yellow, 180 mg; orange, 360 mg; blue, 480 mg).


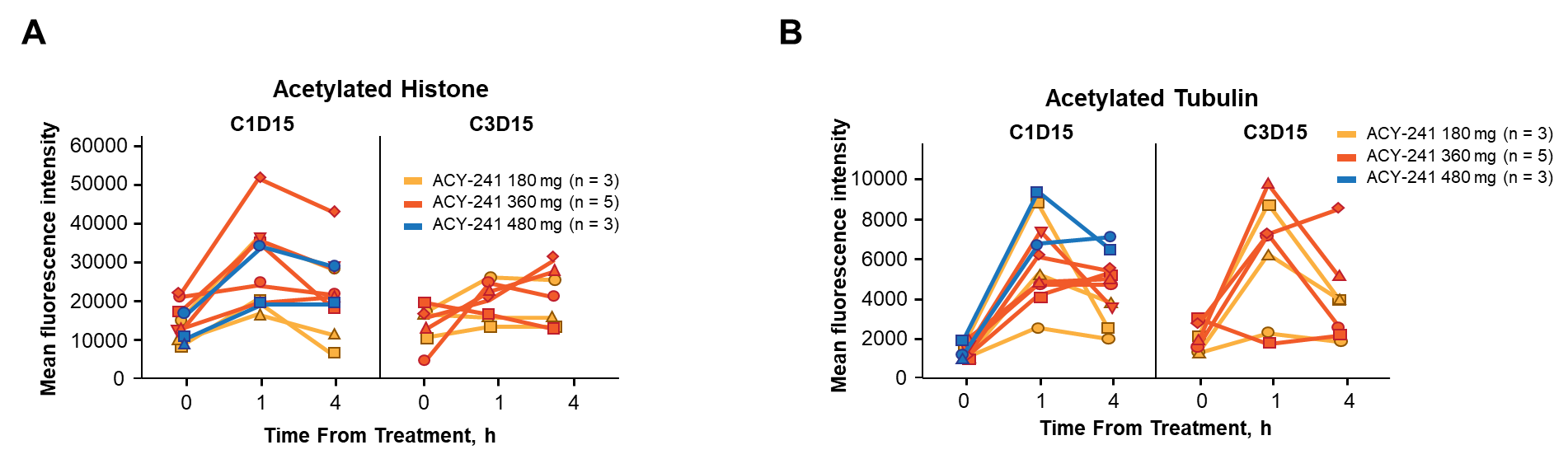


C, cycle; D, day; HDAC, histone deacetylase.

**Supplementary Figure 3.** Circulating cytokine levels under treatment with ACY-241. Amounts of TNF-α **(A)**, IL-1β **(B)**, IL-2 **(C)**, IL-6 **(D)**, IL-4 **(E)**, IL-10 **(F)**, MMP-9 **(G)**, and IFN-γ **(H)** were measured in plasma at screening and indicated visits (C1D15, C3D15). In this representation, individual patients are distinguished by different colors and displayed in subgroups for each of the 3 dose levels (180, 360, and 480 mg).


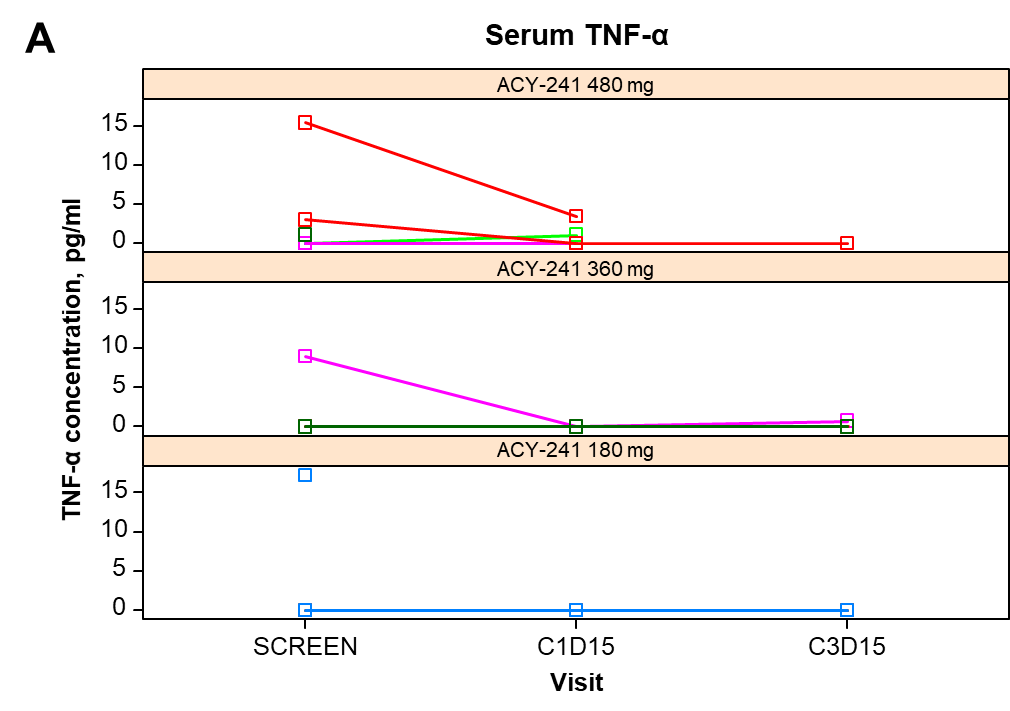


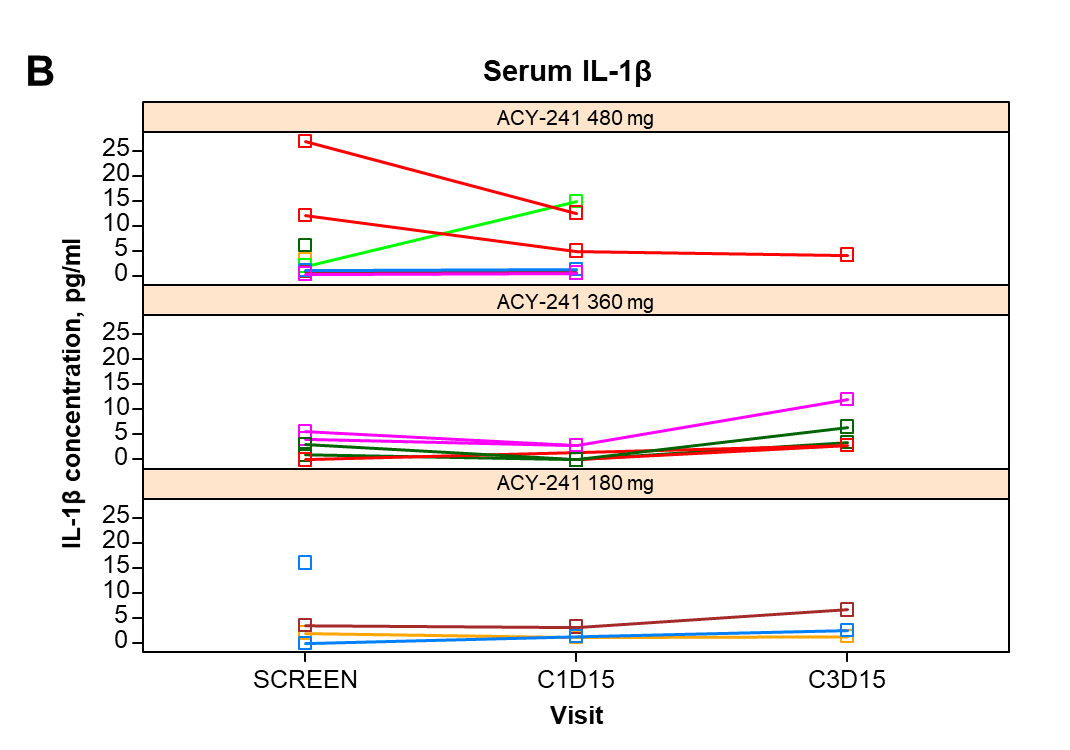

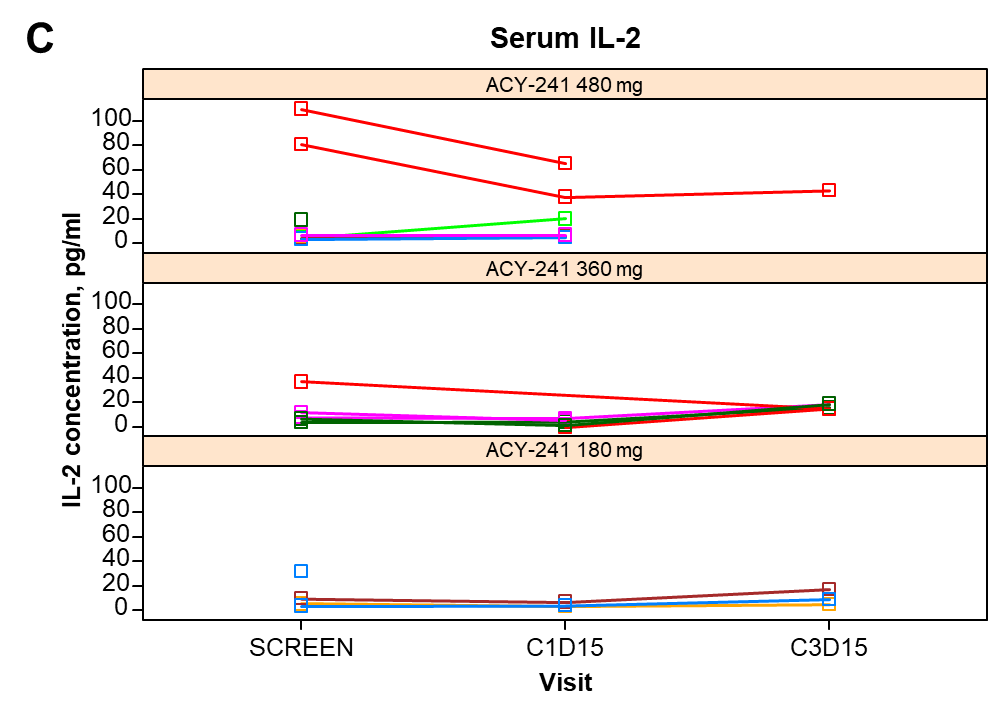

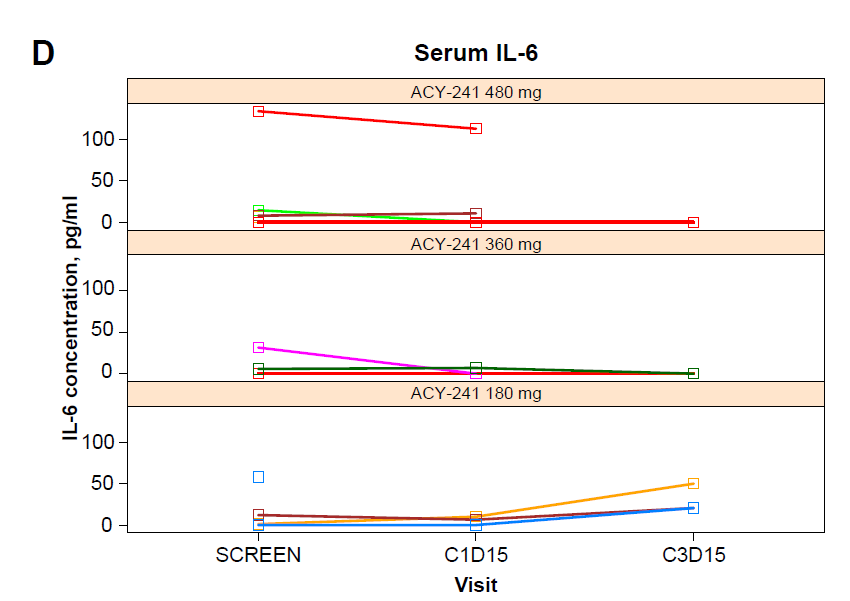


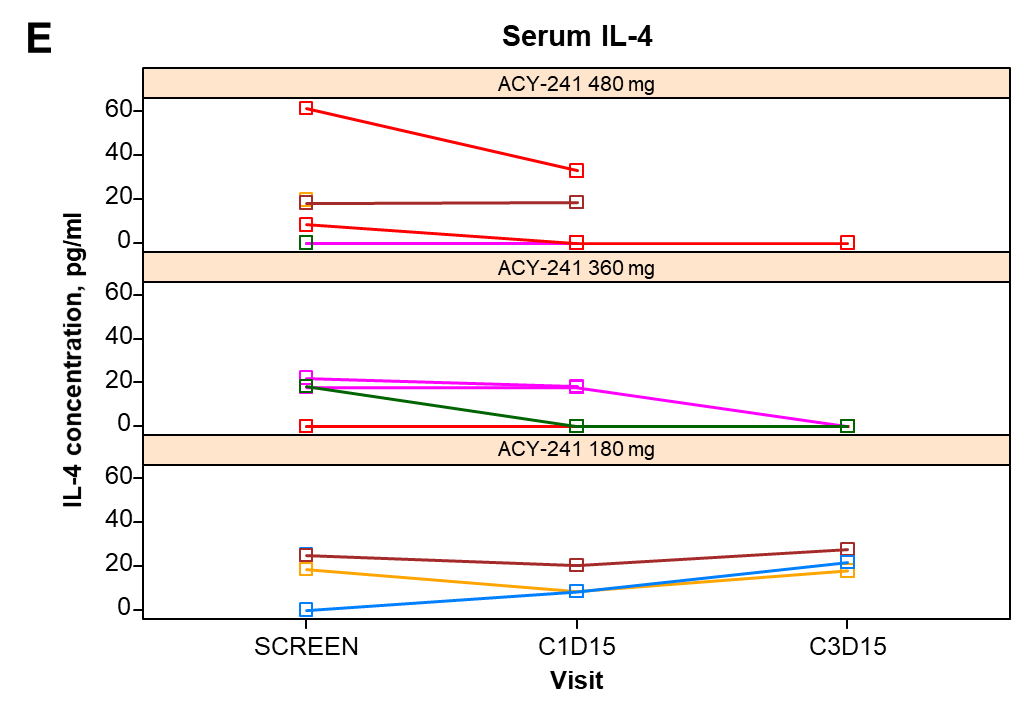


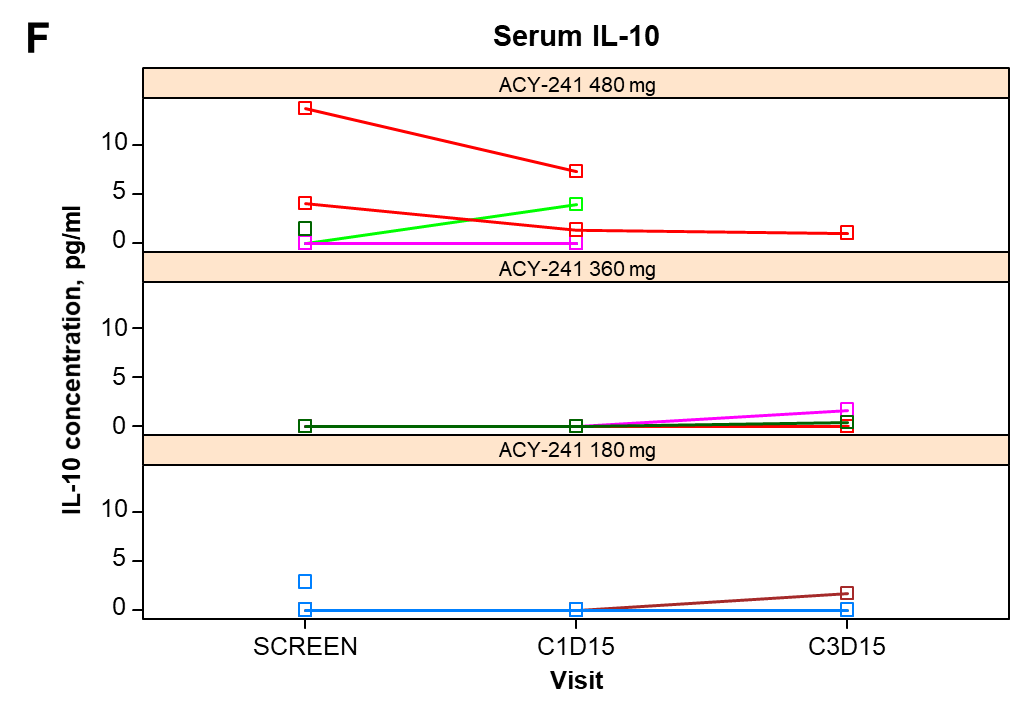


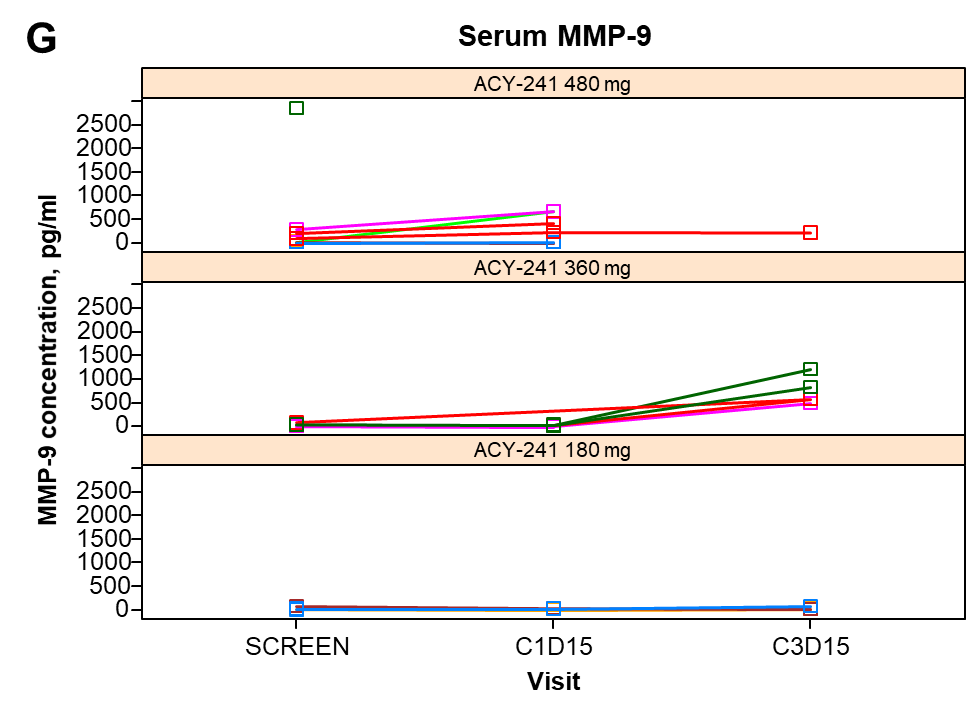


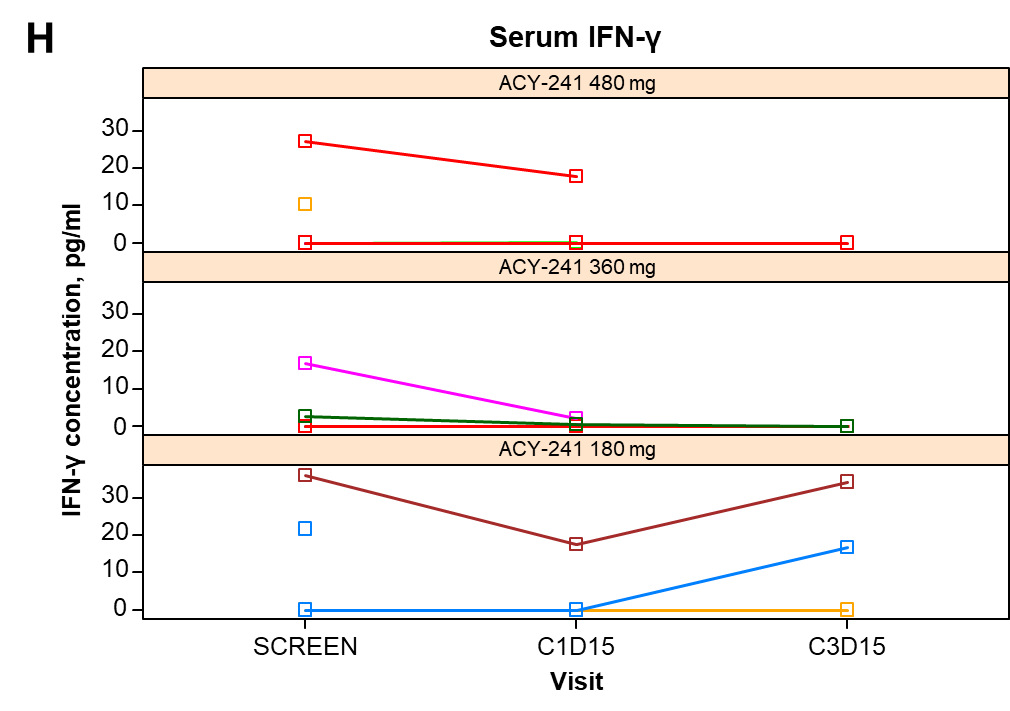


C, cycle; D, day; IL, interleukin; MMP, matrix metallopeptidase.

**Supplementary Figure** **4.** Peripheral immune cell levels: **(A)** peripheral CD4^+^ cells; **(B)** peripheral CD8^+^ cells; **(C)** peripheral CD3^+^ cells; **(D)** peripheral regulatory T cells; **(E)** blood M-myeloid-derived suppressor cells; **(F)** blood G-myeloid-derived suppressor cells; **(G)** naive T helper cells; **(H)** central memory T helper cells; **(I)** effector T helper cells; **(J)** effector memory T helper cells; **(K)** naive cytotoxic T cells; **(L)** central memory cytotoxic T cells; **(M)** effector cytotoxic T cells; **(N)** effector memory cytotoxic T cells, all after ACY-241 treatment. As in Supplementary Figure 3, individual patients (different colors) are grouped by dose level. Percentages of different lymphocyte types are displayed along with the combination of CD markers used to identify specific lymphocytic subpopulations (Tc, Th, Treg, MDSC, and memory T-cell subtypes).


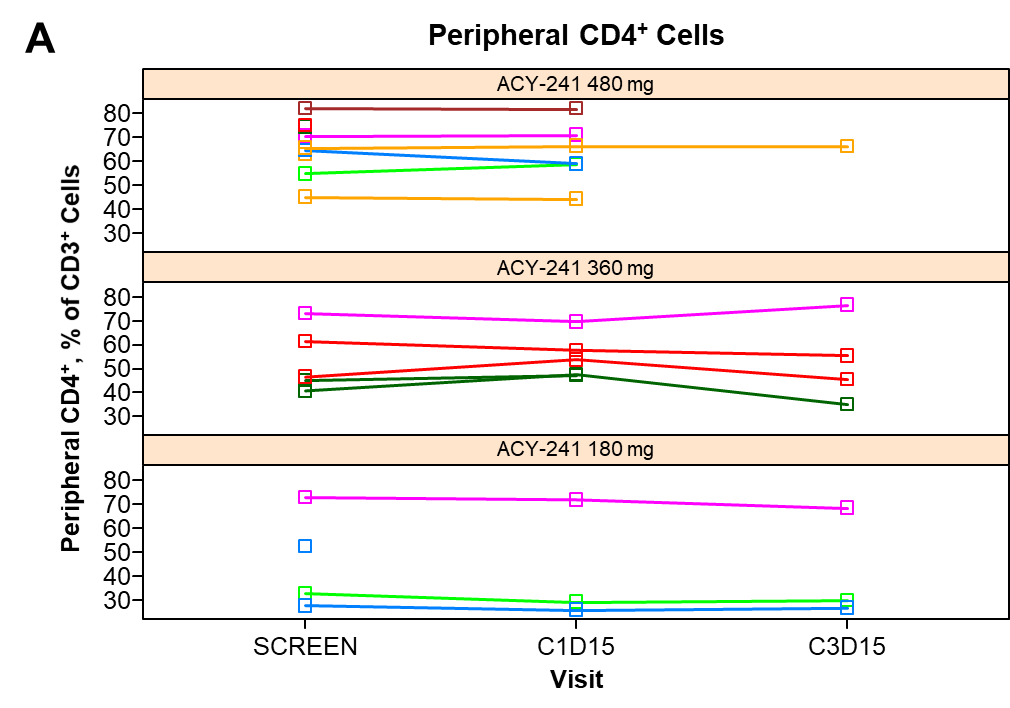


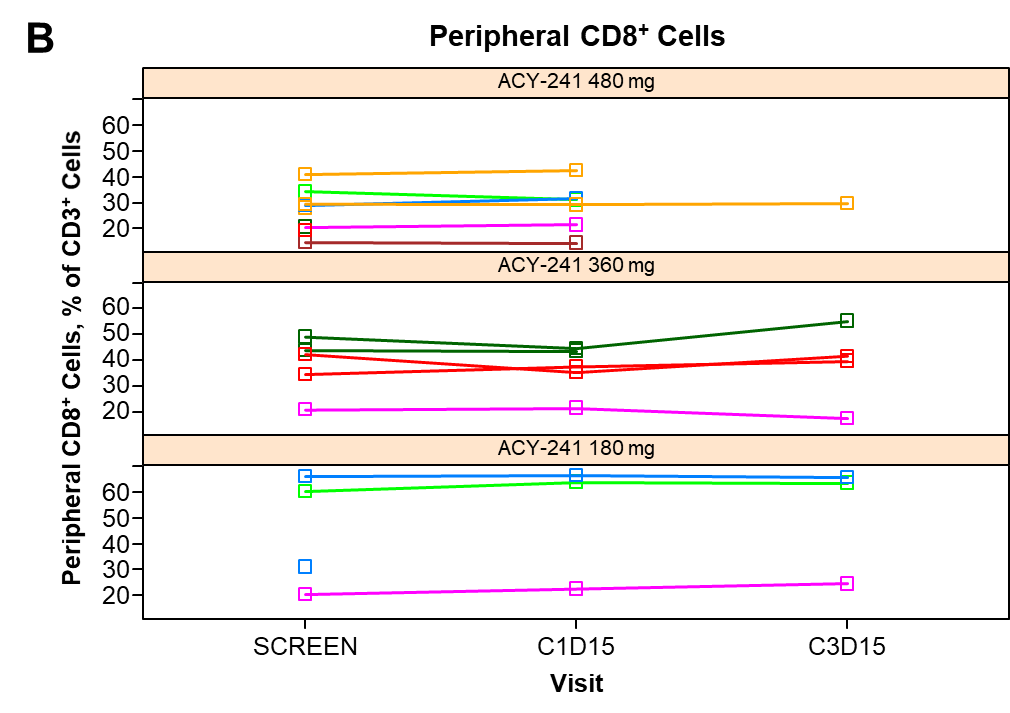


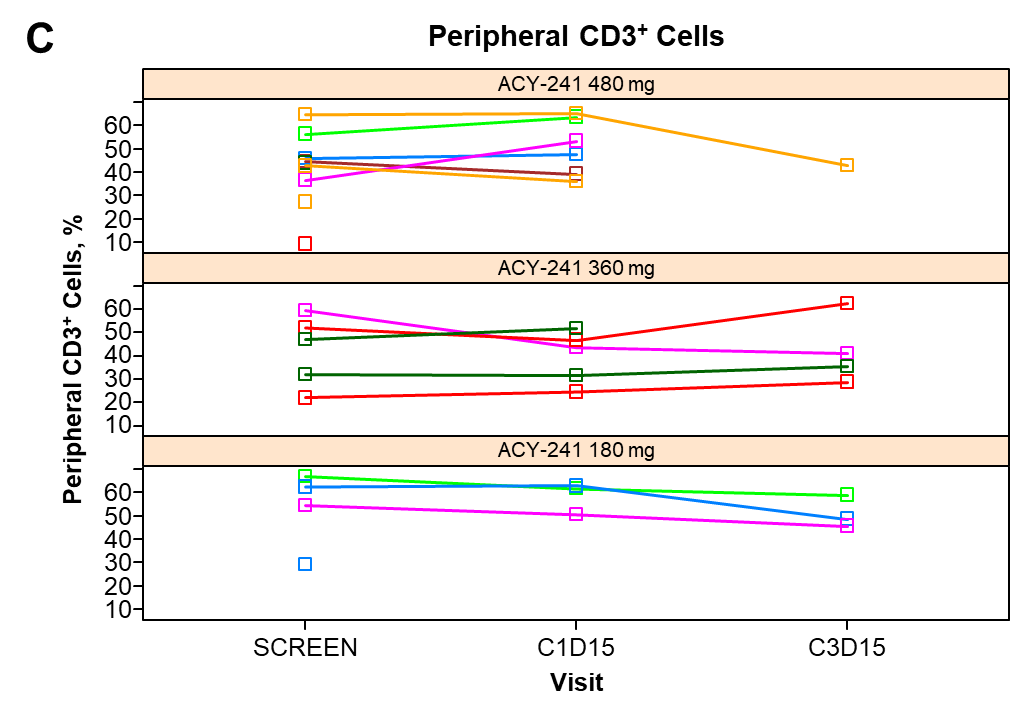


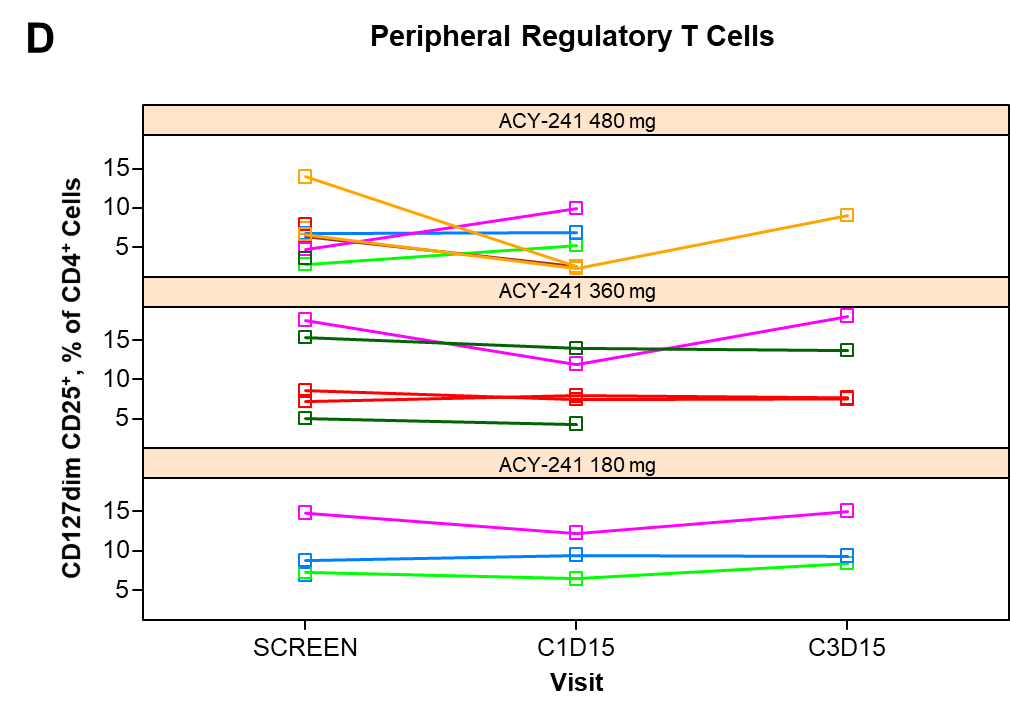


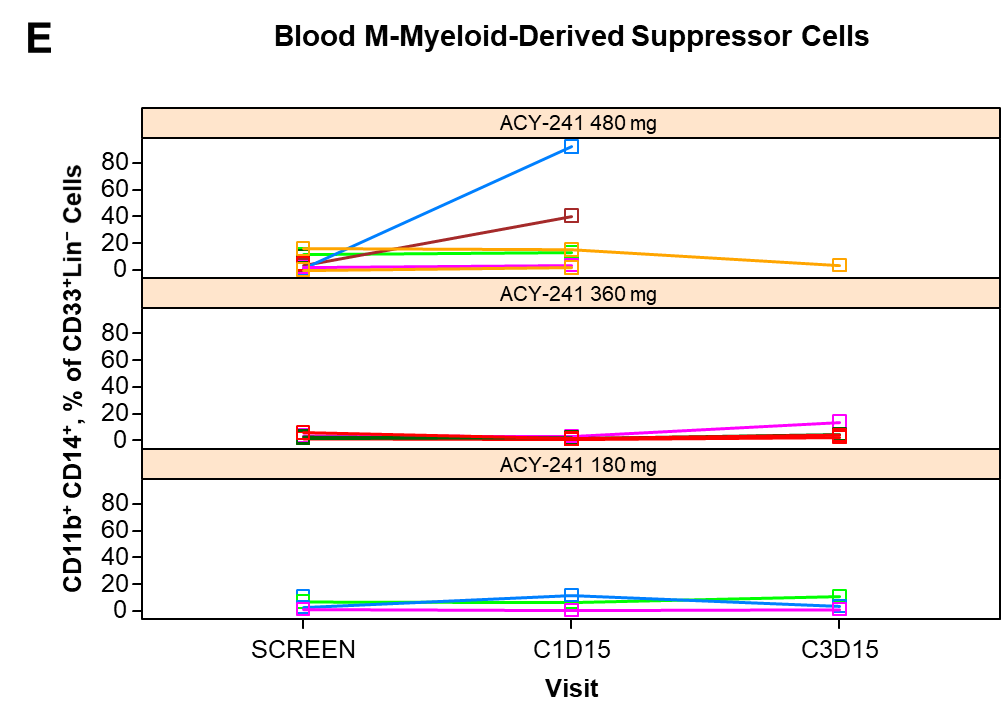


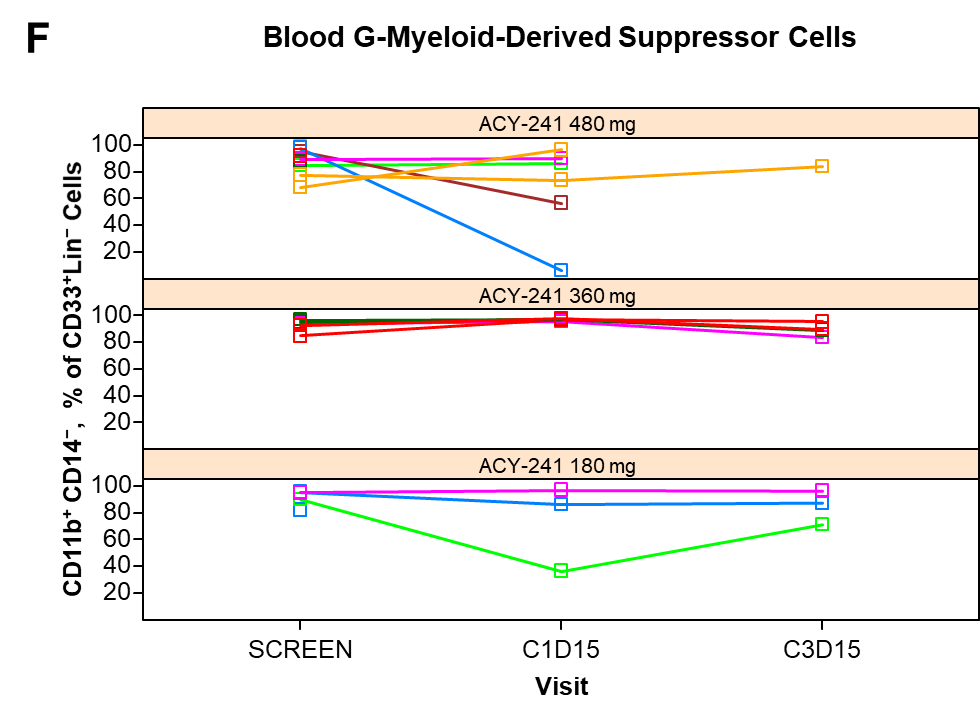


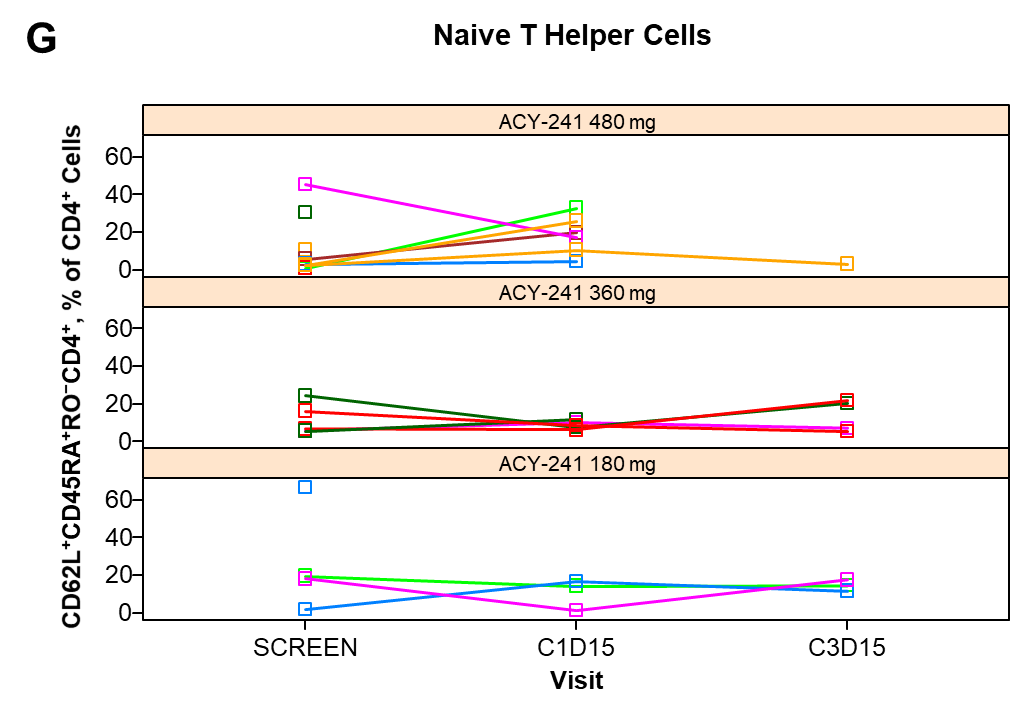


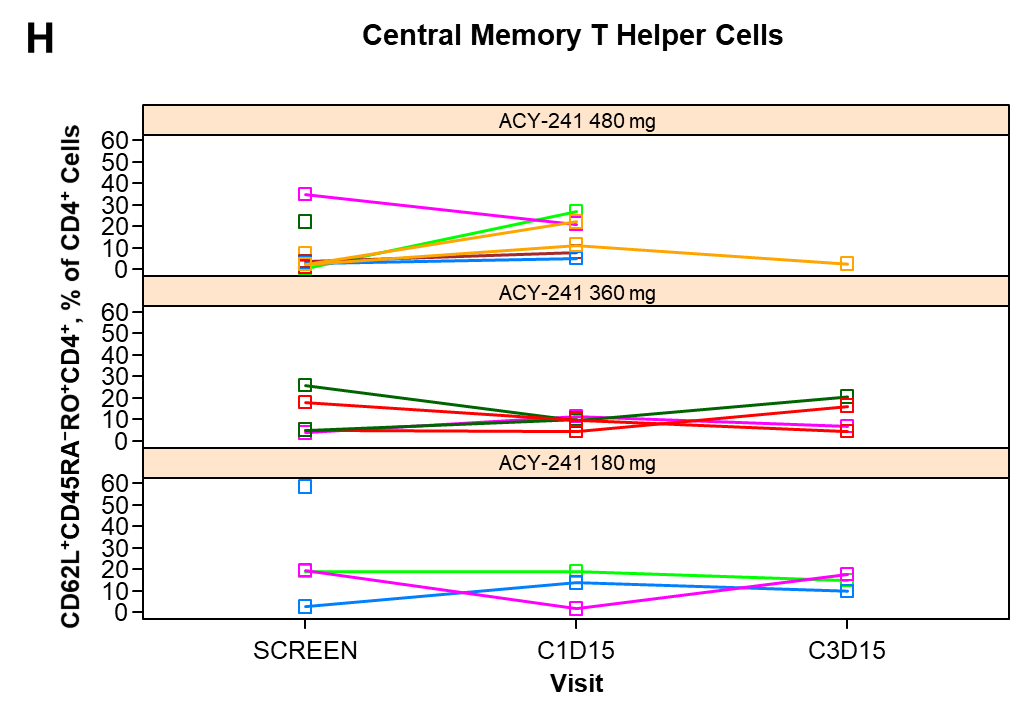


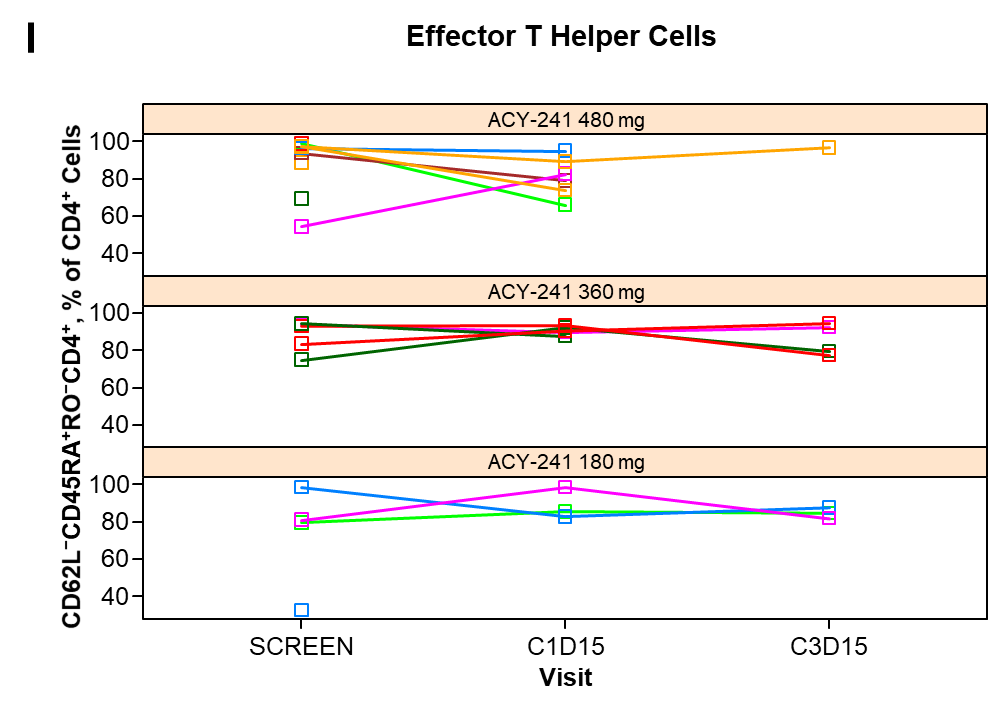


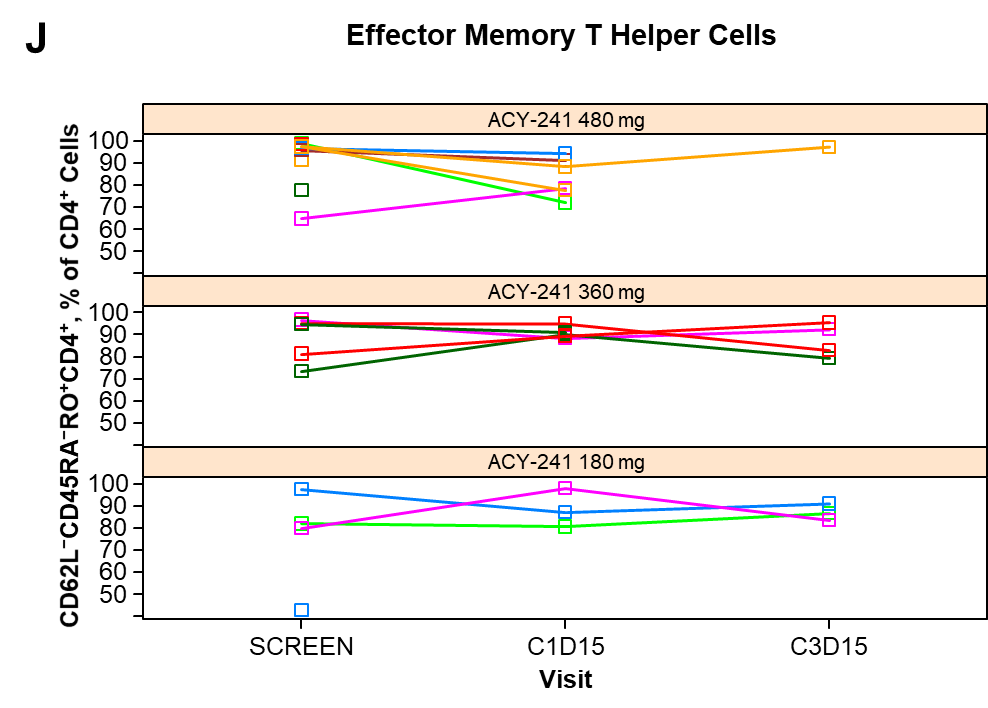


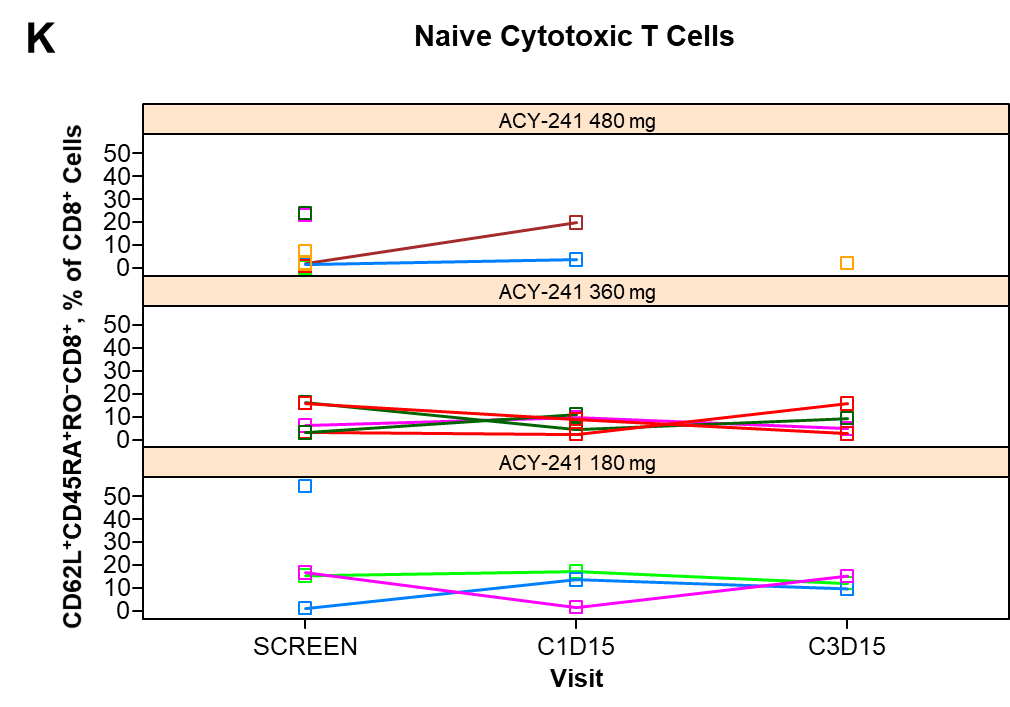


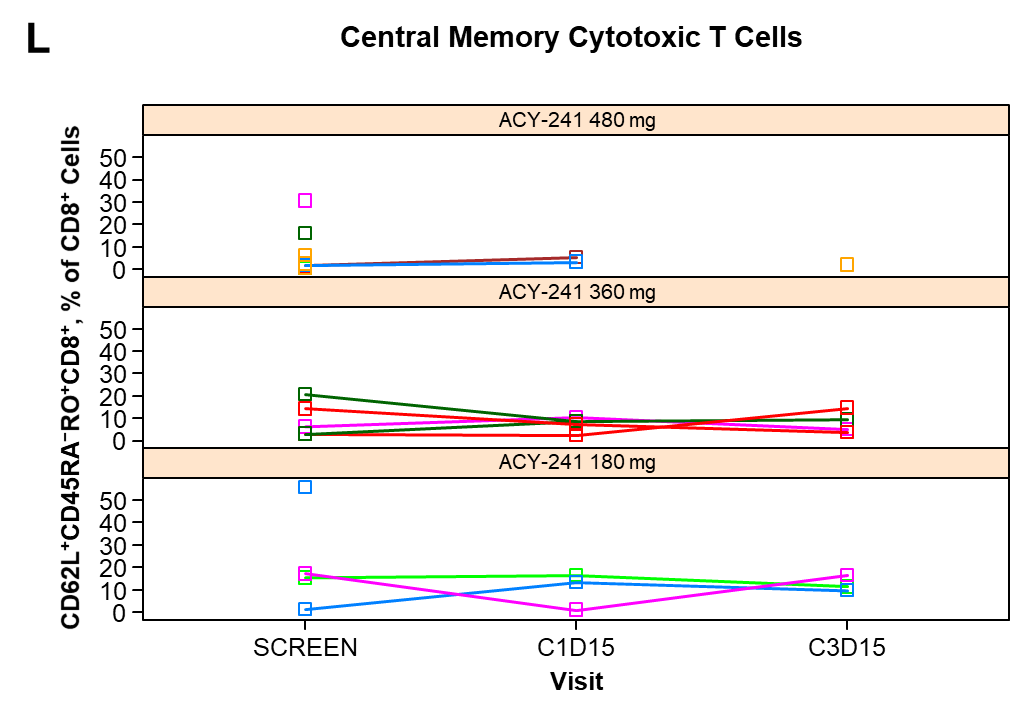


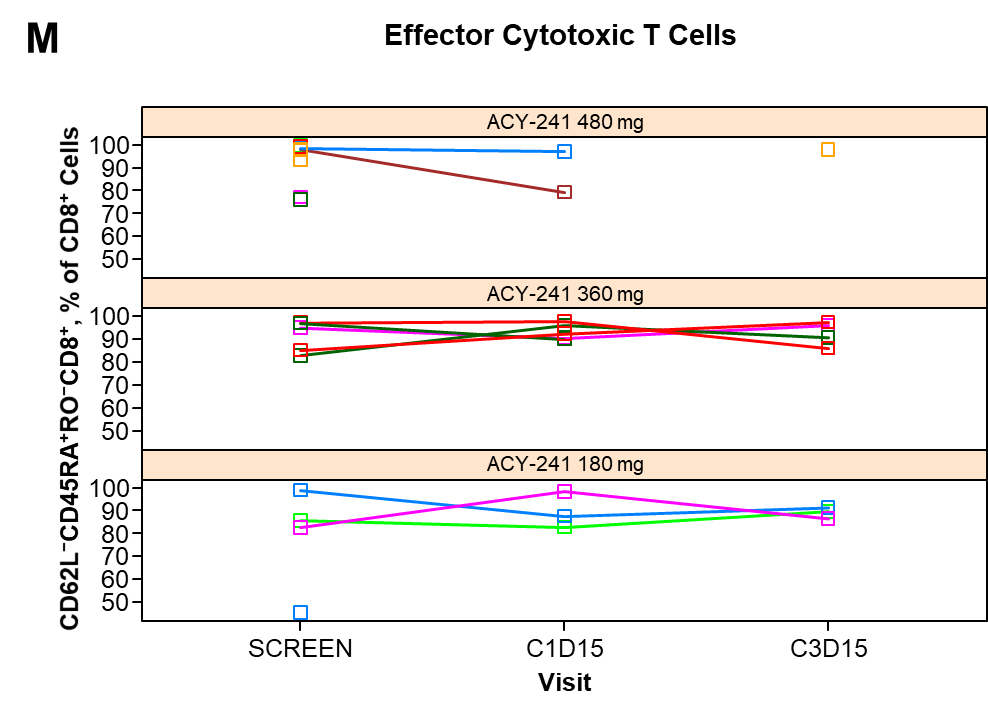


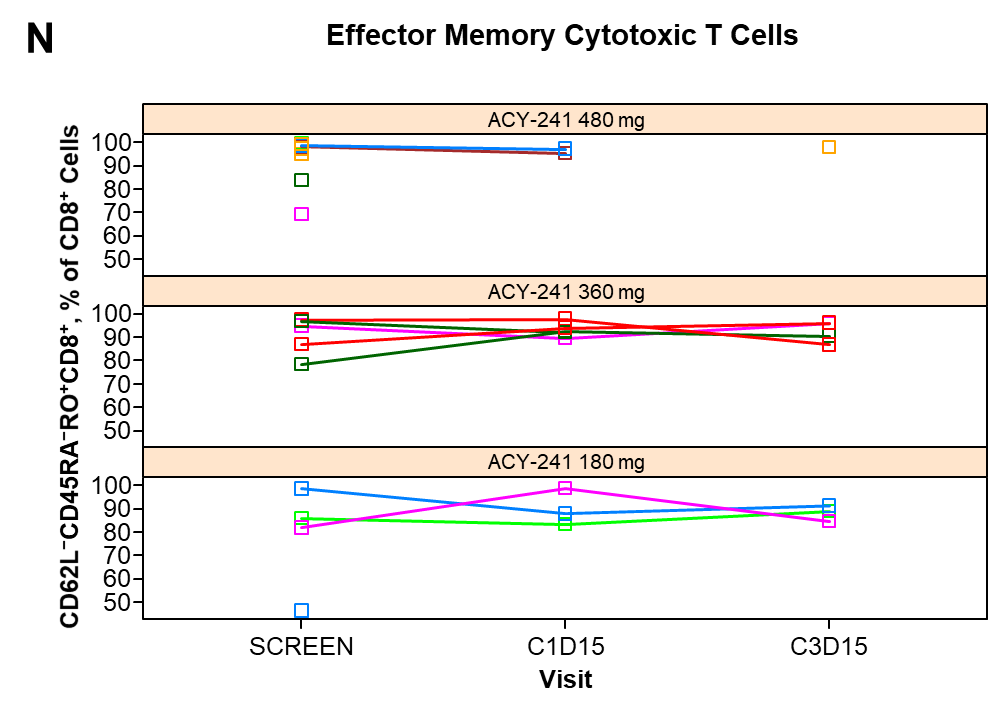


C, cycle; D, day.

**Supplementary Figure** **5.** Tumor-infiltrating cells: **(A)** live single cells; **(B)** CD33^+^ myeloid cells; **(C)** T cell:myeloid cell ratio; **(D)** CD4^+^ T cells; **(E)** CD4:CD8 T cell ratio; **(F)** CD4^+^ PD-1^+^; **(G)** CD8^+^ T cells; **(H)** CD8^+^ PD-1^+^; **(I)** regulatory T cells; **(J)** CD56^+^ NK cells; **(K)** Ki-67^+^ NK cells; **(L)** CD56^+^ CD3^+^ NK T cells. Percentages of immune cell subpopulations (not already represented in Figure 5) for individual patients in tumor biopsies obtained before and after treatment with ACY-241. The 2 different dose levels to which these patients were assigned are indicated by different colors (orange, 360 mg; blue, 480 mg).


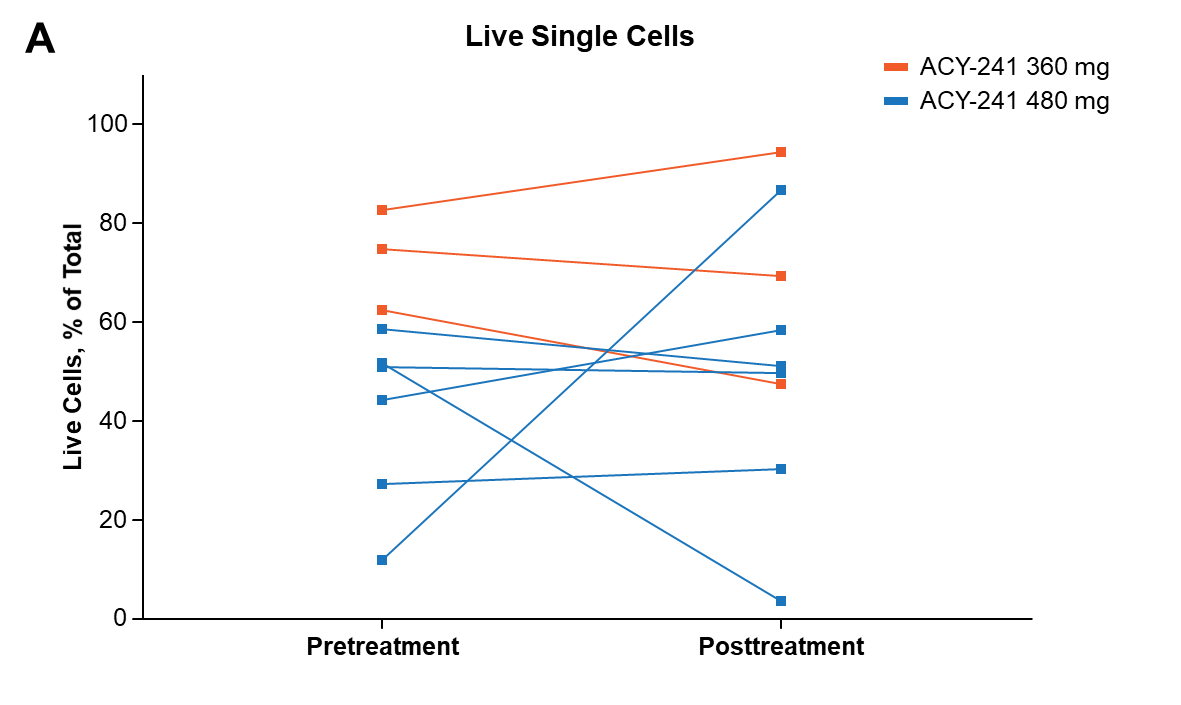


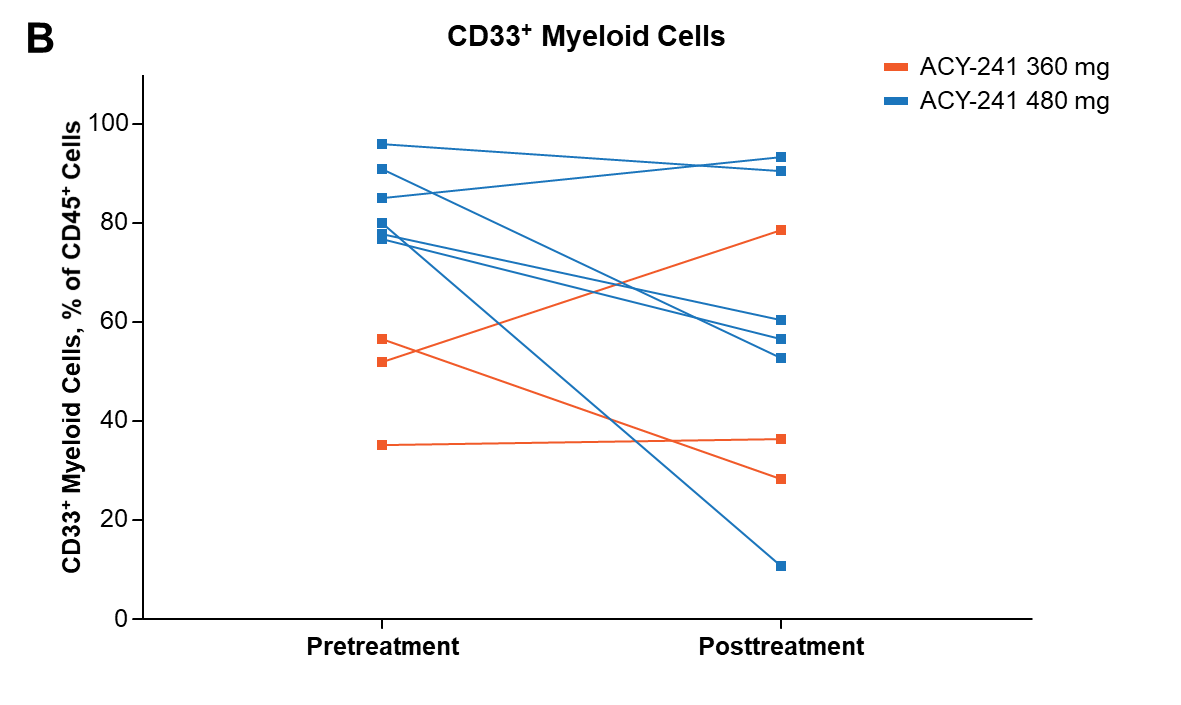


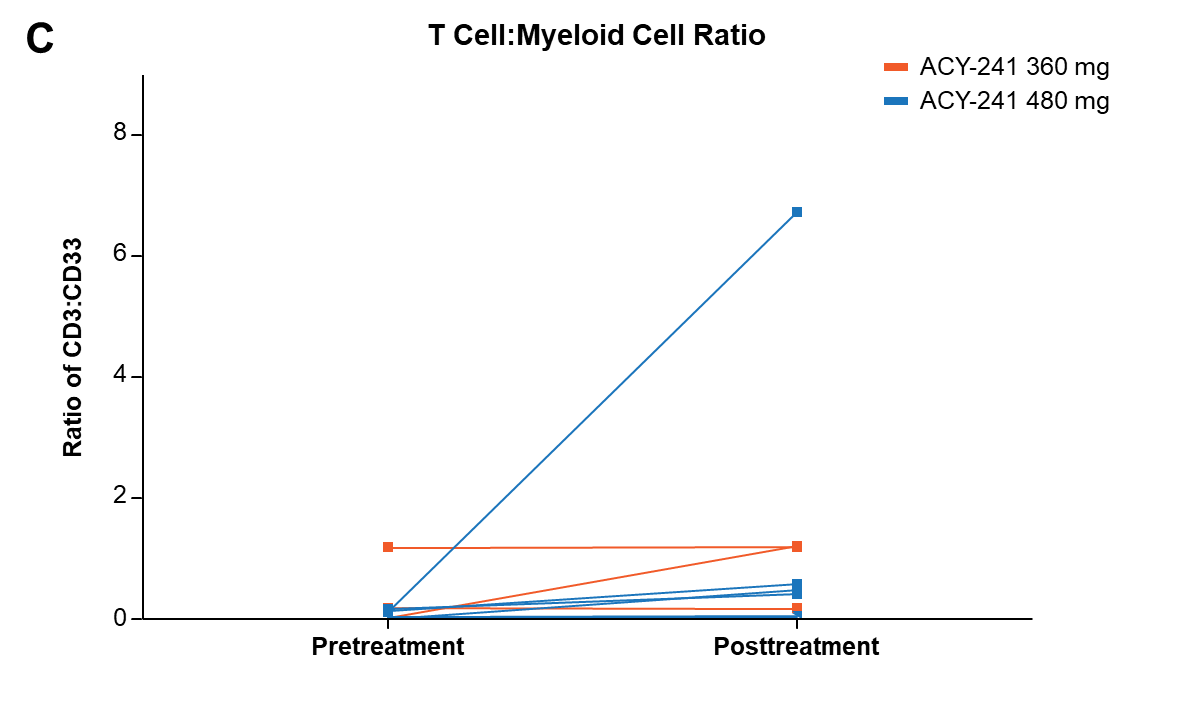


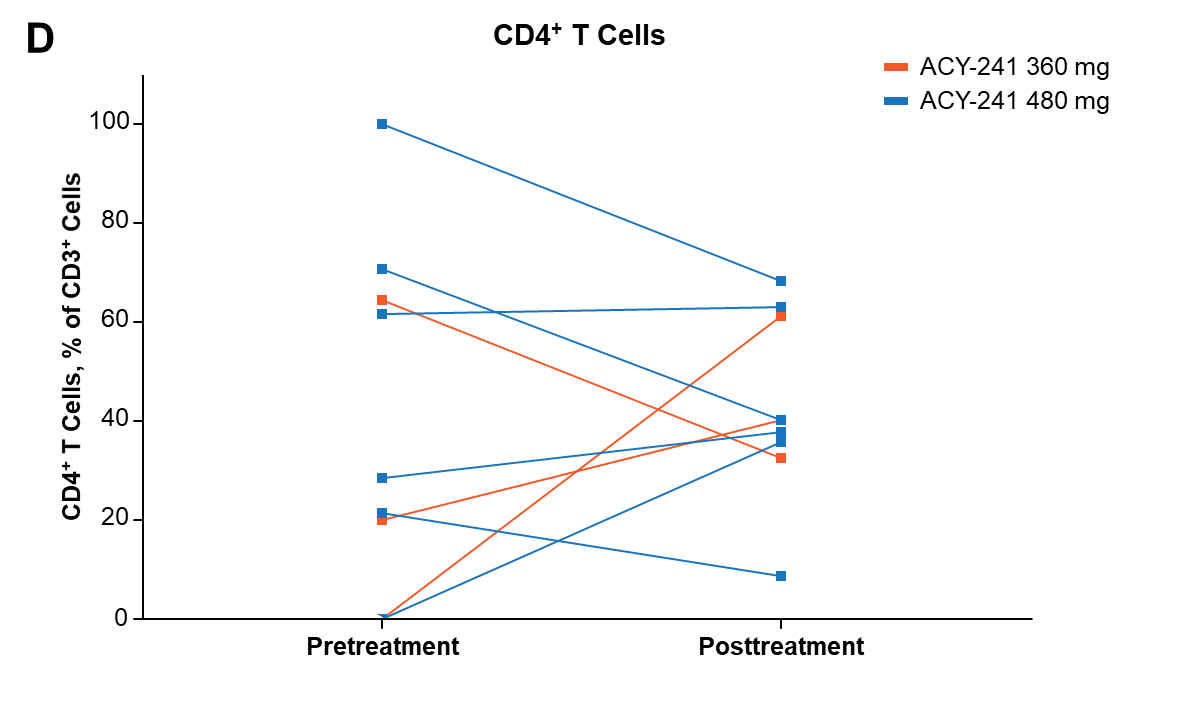


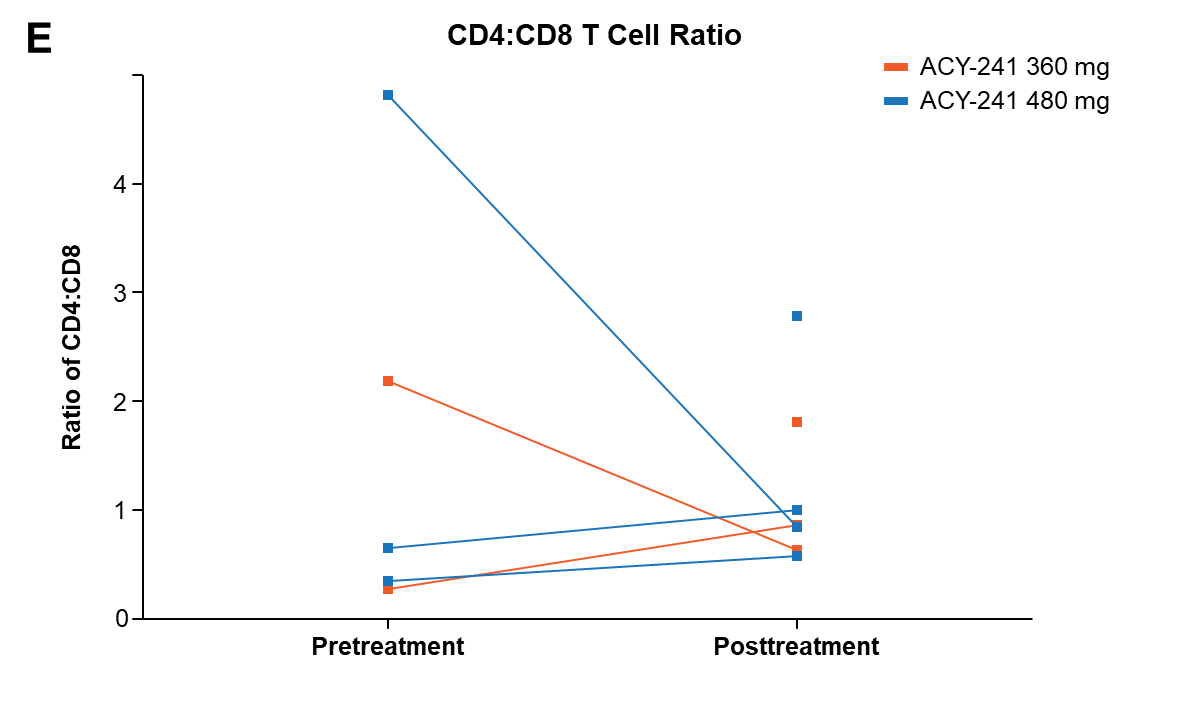


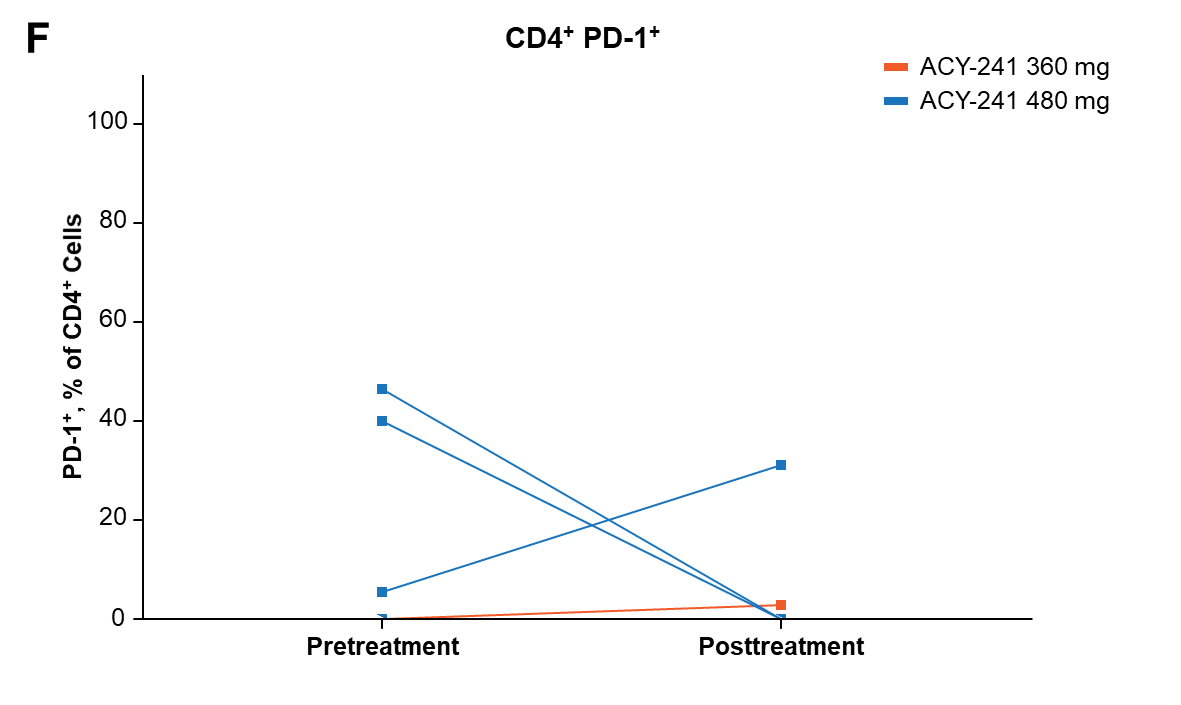


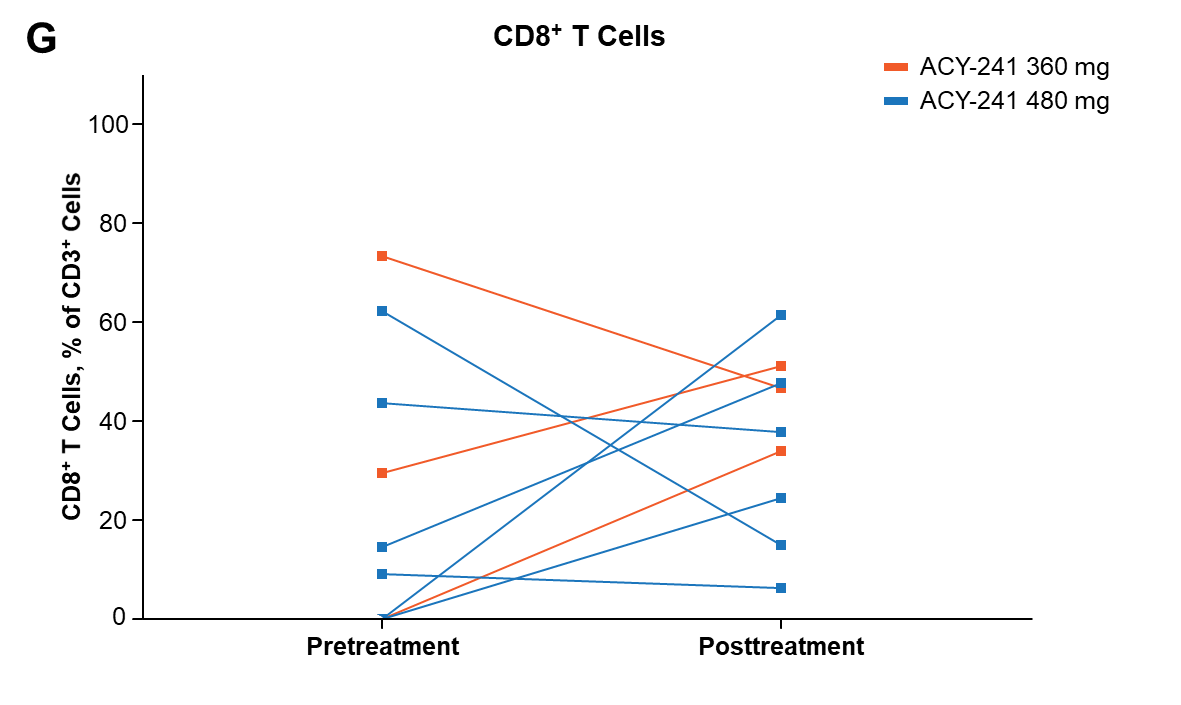


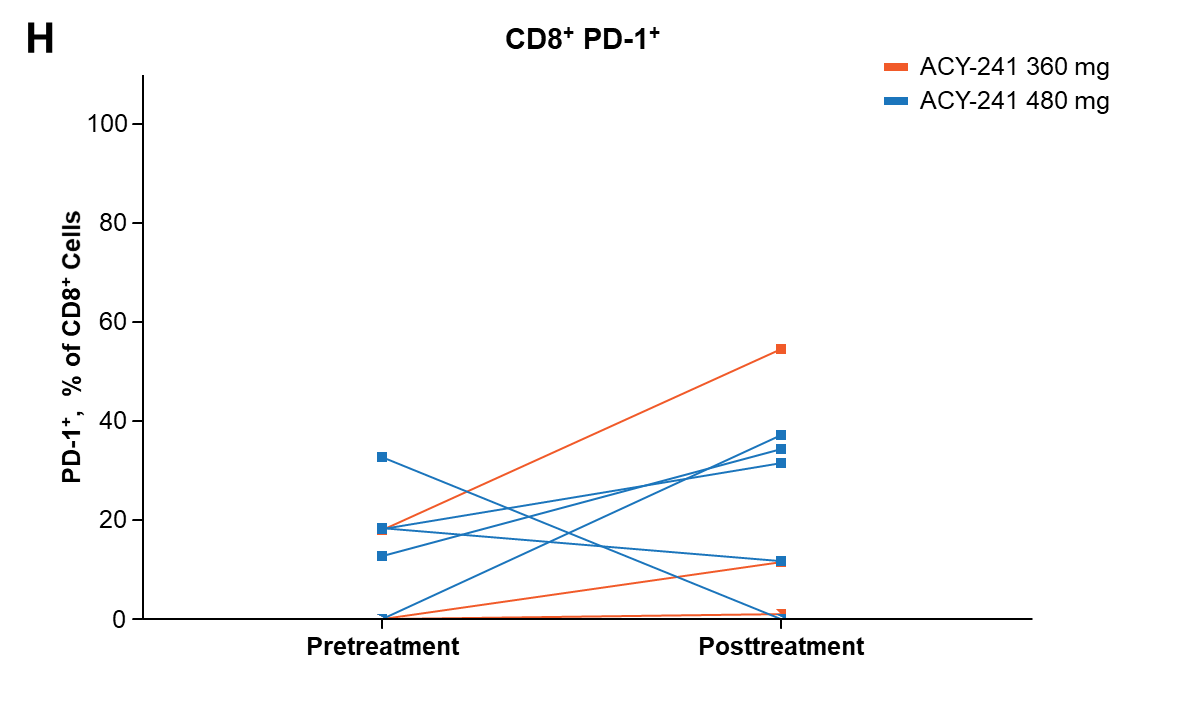


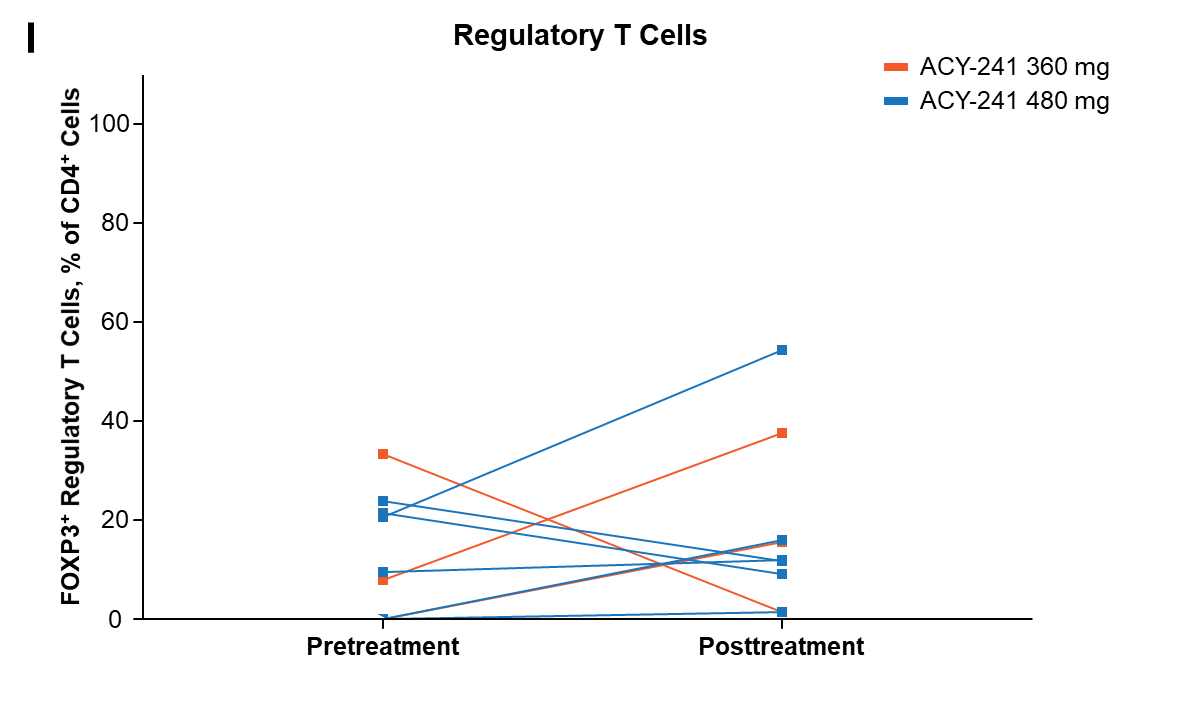


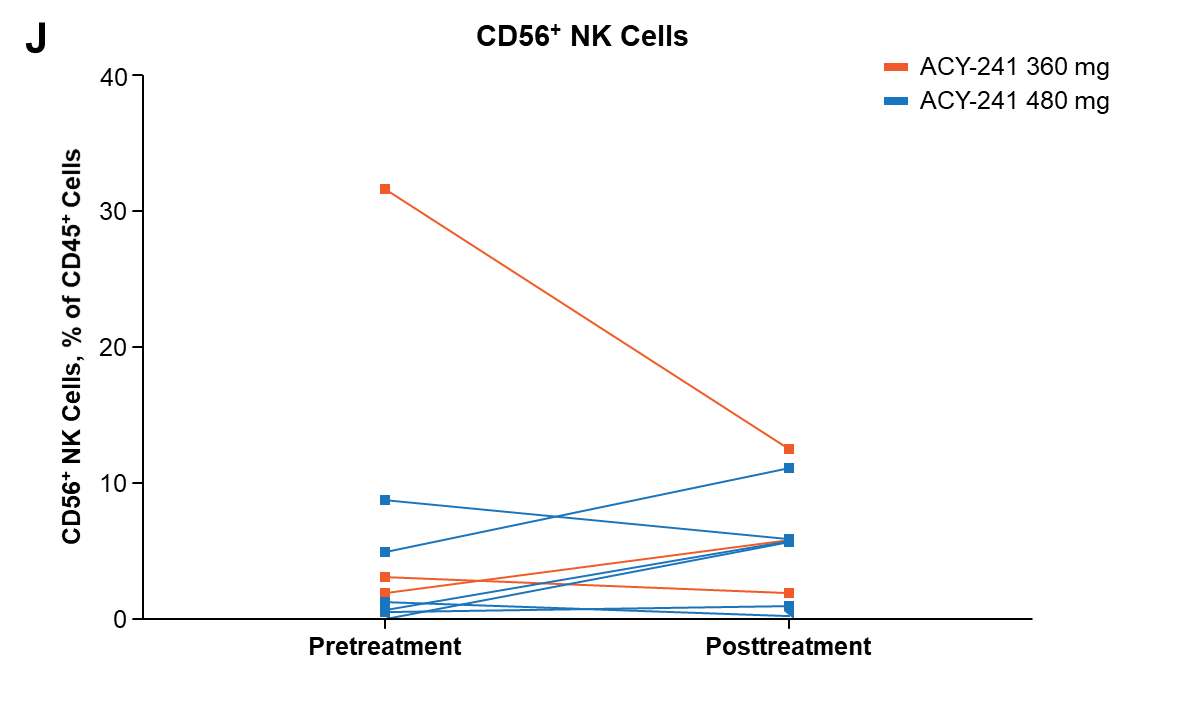


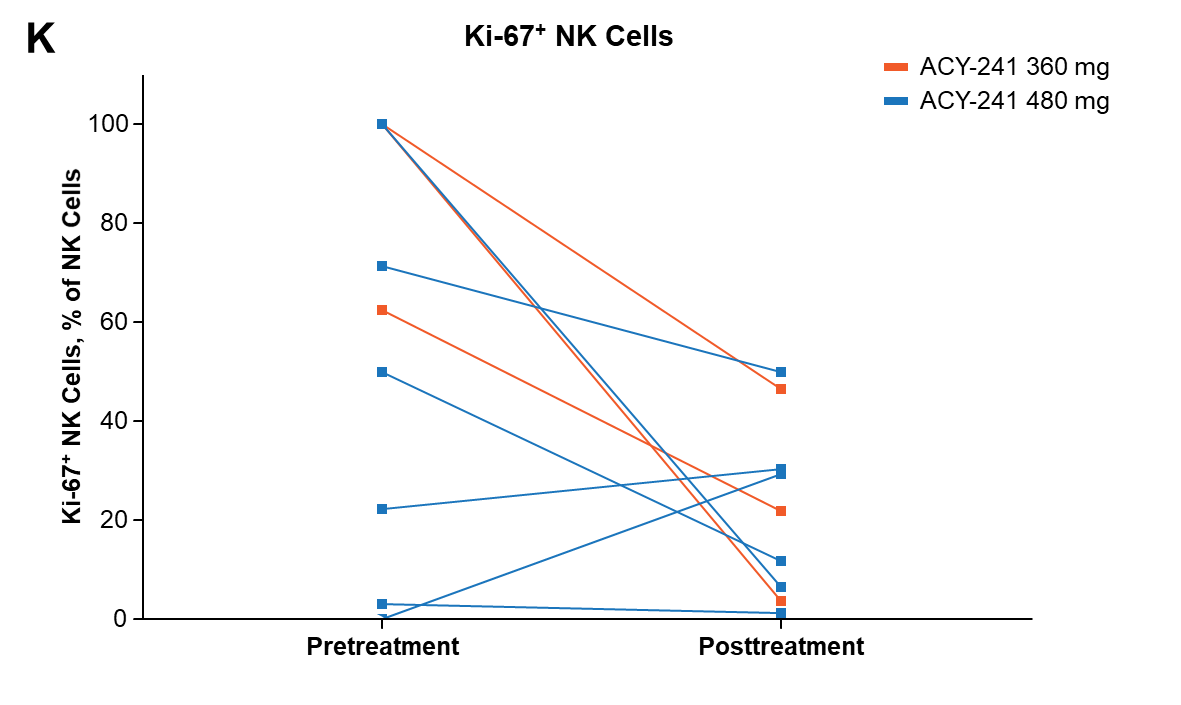


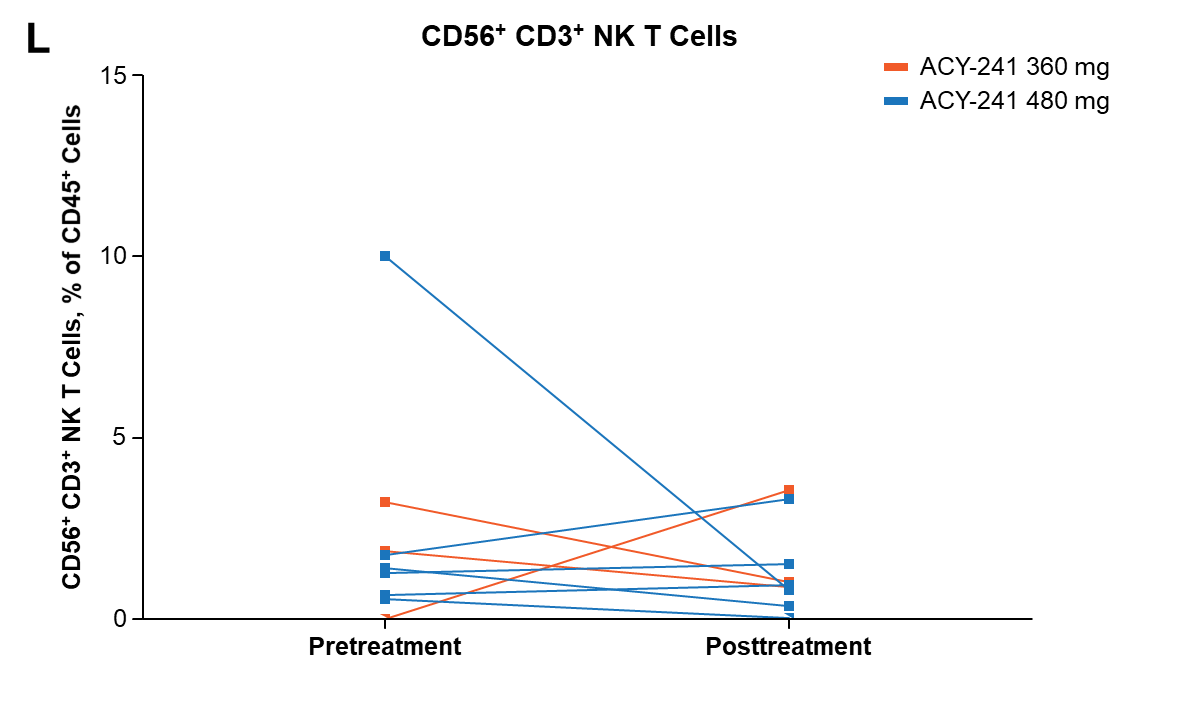


NK, natural killer; PD-1, programmed death-1.
